# Diagnosis Documentation Done Right: Cross-Specialty Standard for the Diagnosis Section in German Discharge Summaries — A Mixed-Methods Study

**DOI:** 10.1007/s11606-025-09395-9

**Published:** 2025-02-06

**Authors:** Julian Frings, Paul Rust, Sven Meister, Christian Prinz, Leonard Fehring

**Affiliations:** 1 Faculty of Health, School of Medicine, Witten/Herdecke University, Witten, Germany; 2 Health Care Informatics, Faculty of Health, School of Medicine, Witten/Herdecke University, Witten, Germany; 3 Department Healthcare, Fraunhofer Institute for Software and Systems Engineering ISST, Dortmund, Germany; 4 Helios University Hospital Wuppertal, Department of Gastroenterology, Witten/Herdecke University, Wuppertal, Germany

**Keywords:** Electronic discharge summary, Clinical documentation standards, Diagnosis section, Physician preferences, Structure and content, Mixed-methods research

## Abstract

**Background:**

The diagnosis section in hospital discharge summaries is critical for continuity of care and patient safety, yet it varies widely in quality, format, and content due to a lack of standards.

**Objective:**

This study aims to develop a cross-specialty standard for the structure and content of the diagnosis section, based on the preferences of German physicians. The study examines physicians﻿’ satisfaction with the diagnosis section, their rating of its importance, and their preferences for its specific elements, comparing perspectives between inpatient and outpatient physicians.

**Design, Participants, Approach:**

This mixed-methods study integrated a scoping review, focus group discussion, and a nationwide survey of 602 physicians (317 outpatient primary care and 285 inpatient physicians; 4.1% response rate), most trained in internal medicine. Quantitative analyses evaluated physician satisfaction and preferences, while qualitative feedback provided deeper insights regarding preferred content and format.

**Key Results:**

Although 95.7% of physicians considered the diagnosis section crucial for follow-up care, only 36.9% were satisfied with its current content and format. 91.2% supported standardizing the diagnosis section, identifying 18 content elements to be included for every current treatment diagnosis. Strong consensus (> 95.0% agreement) was reached for “name of the diagnosis,” “severity/stage/classification/TNM,” “localization/extent/pattern of involvement,” “course e.g., acute, chronic, recurrent,” “expression,” “complications,” “date of initial diagnosis,” and “etiology/cause.” 86.4% preferred separating current and chronic/prior diagnoses with headings. Outpatient physicians were more likely than inpatient physicians to rate “ICD-10 codes” as mandatory (46.2% vs. 14.8%, *p* < 0.001) and to consider “recommendations for further procedures” (76.6% vs. 63.6%, *p* < 0.001) and “follow-up appointments” (77.3% vs. 63.5%, *p* < 0.001) as necessary. Additionally, a list of practical recommendations for clinicians to better document diagnoses was derived.

**Conclusions:**

This study proposes a cross-specialty standard for the diagnosis section based on physician preferences for a clearly structured format and 18 key content elements.

**Supplementary Information:**

The online version contains supplementary material available at 10.1007/s11606-025-09395-9.

## INTRODUCTION

Healthcare’s digital transformation is redefining patient care by enhancing patient safety,^1^ improving clinical efficiency,^2^ and enabling data-driven research and decision-support.^3^ Driving this transformation is the adoption of digital documentation systems, such as electronic health records (EHRs) and electronic discharge summaries, which reshape how healthcare providers manage and share patient information. However, their implementation faces significant barriers,^4^ including poor interoperability,^5,6^ misalignment with clinical workflows,^7^ and increased administrative burden,^8^ which together undermine efficiency and user satisfaction.^9^ Often, physicians are insufficiently involved in designing documentation systems, despite being their primary users.^10^ The American Medical Association has identified poorly designed documentation systems as a major contributor to physician burnout,^11^ highlighting the urgent need for user-centered design approaches that involve physicians in setting clinical documentation standards.^12^

Discharge summaries, the most important communication tool between inpatient and outpatient physicians, are particularly affected by a lack of standardization. Research consistently shows that discharge summaries suffer from significant variability in quality,^13,14^ content,^15–17^ format,^18,19^ and timeliness,^20,21^ which can lead to miscommunication and comprise both patient safety and continuity of care.^14,22^ Additionally, the lack of standardized documentation impedes interoperability, obstructs seamless health information exchange, complicates information retrieval, and hampers digital health innovations like clinical decision support or real-word evidence studies.^23,24^

Despite efforts to standardize discharge summaries, including the guidelines set forth by the eHealth Network of the European Union (EU) in November 2023,^25^ there remains limited understanding of physicians’ preferences, particularly concerning the structure and content of the diagnosis section. This section, alongside the medication plan, is crucial for physicians.^26,27^ While some countries, like Germany, have standardized the medication plan in discharge summaries, the diagnosis section remains largely unstandardized, lacking a consistent format and clear guidelines on the essential content elements for documenting diagnoses. Unsurprisingly, physicians are dissatisfied with the diagnosis section: for instance, a UK study found that over half of diagnosis sections contained inaccurate diagnoses,^28^ while a survey of German outpatient physicians revealed that 96.4% frequently encountered ambiguous discharge documentation.^29^ Outpatient physicians often struggle to find salient information due to confusing layouts ^30^ and missing information, such as clear and accurate diagnoses and ICD-10 codes.^31,32^

The Health Level 7 (HL7) Clinical Document Architecture (CDA) serves as an international technical standard that defines the structure and semantics of clinical documents for electronic exchange and is widely adopted in electronic documentation systems worldwide.^33^ In its current form, the CDA diagnosis section is limited to entry fields for admission and discharge diagnoses, corresponding ICD-10 codes, localization, and free-text.^33^ However, this minimal structure often fails to meet clinical needs, where the diagnosis section typically contains additional elements, such as a brief summary of the patient’s disease course and medical history, akin to the problem list found in many EHR systems. Therefore, this study aims to develop a standard for the structure and content of the diagnosis section in discharge summaries, based on the preferences of German physicians and applicable across medical specialties. To achieve this, the following research questions (RQ) will be addressed:

- RQ1: How important is the diagnosis section to physicians, and how satisfied are they with its current state?
- RQ2: Do physicians support the establishment of structural and content standards for the diagnosis section?
- RQ3: What content and structural elements do physicians prefer for the diagnosis section?
- RQ4: How do preferences for content and structure differ between inpatient and outpatient physicians?

## DESIGN, PARTICIPANTS, AND APPROACH

### Study Design

This study followed a three-step approach. First, a scoping review was conducted to gather existing research on physicians’ preferences for the diagnosis section and inform the development of both the survey and focus group discussion (FGD). Second, a FGD with experts on discharge documentation was held to gain qualitative insights and further refine the survey. Lastly, an online survey was administered to a broad, nationwide sample of German physicians, capturing their ratings of importance, satisfaction, and preferences regarding the structure and content of the diagnosis section.

This study was reviewed by the ethics committee of the Witten/Herdecke University (number, S-311/2023) and no ethical or professional concerns were raised.

### Literature Review

We conducted a scoping review following the PRISMA-ScR guidelines,^34^ searching PubMed, Scopus, Cochrane, and MEDLINE databases, supplemented by manual searches using a search engine and bibliographies (snowball method). A search strategy with 32 search terms, no date restrictions, and five predefined inclusion criteria was applied. The PRISMA-ScR checklist, search terms, and inclusion criteria are detailed in Supplementary Table 1 and 2 and Supplementary Note 1.

**Figure 1:**
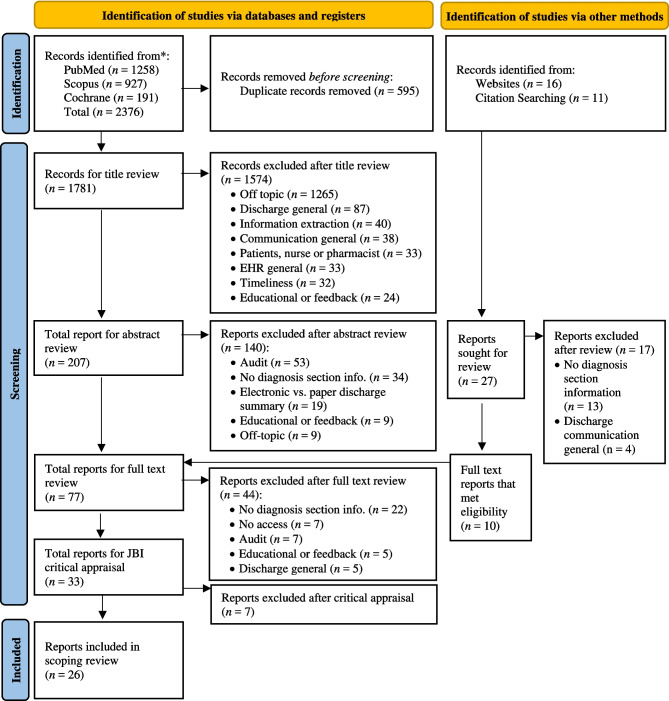
The flowchart shows the sequential screening process during the literature scoping review.^90^

A PRISMA flow diagram (Fig. 1) summarizes the study selection process. Supplementary Table 3 provides a sample of excluded articles by reason. Methodological quality was assessed using the Joanna Briggs Institute (JBI) critical appraisal tools.^35–38^ Critical appraisal scores for included articles are provided in Supplementary Table 4.

Data from the selected articles was extracted, including study details, findings related to the diagnosis section, and whether a template was developed. The literature was synthesized into key themes, discussed with the focus group, and used to inform the survey design.

### Focus Group Discussion

To validate the findings from the literature scoping review and inform survey development, a FGD was conducted following the COREQ checklist (see Supplementary Table 5).^39^ A structured guide (see Supplementary Note 2) was used during the FGD, where participants were presented with lists of structural and content elements identified in the scoping review. They were asked to expand these lists based on their experience and validate their completeness, which were then used to assess physician preferences in the subsequent survey. Additionally, participants were asked to refine and validate the survey answer options related to reasons for insufficient diagnosis sections. Participants were selected via purposive sampling, based on their expertise in discharge documentation, demonstrated through teaching, publications, contributions to digitizing discharge documentation, or over a decade of experience reviewing and signing off discharge summaries.^40,41^ The FGD included five inpatient and two outpatient physicians, all from different institutions. The virtual session was video-recorded and transcribed for qualitative analysis in MAXQDA software (version 24.2).^42^ The transcript was analyzed by JF and LF to identify key themes related to preferences for diagnosis documentation and implications for the survey design.^43^

### Online Survey

We next conducted a survey investigating satisfaction with the diagnosis section and preferences of German physicians on its structure and content. The survey design and reporting adhered to the CHERRIES checklist for online surveys (see Supplementary Table 6).^44^

#### Participant Selection and Survey Administration

We used a modified Dillmann technique^45^ to recruit physicians via two channels. Outpatient physicians were contacted via email through regional chapters of the General Practitioners’ Association and cooperating Associations of Statutory Health Insurance Physicians. Inpatient physicians were recruited via the Helios Healthcare mailing list, the largest hospital network in Germany, encompassing 87 hospitals.^46^ Based on a predicted 3–5% response rate from similar surveys,^47^ we contacted 14,845 physicians, achieving an overall response rate of 4.1%. The survey, administered via LimeSurvey (version 6.4), ran from April to July 2024. No incentivization was provided.

#### Survey Questionnaire

The survey questionnaire was developed based on insights from the literature review and FGD. After drafting, it was pretested (see Supplementary Table 6, item 5).

The final survey comprised 31 questions across five sections: (1) use of the diagnosis section (2), satisfaction with and importance of the diagnosis section, (3) content preferences, (4) structural preferences, and (5) demographic and professional background. Participants were informed of the research objectives, expected completion time, ethics committee approval, and data privacy policy before providing informed consent. Survey responses were recorded anonymously.

Sections (1) and (2) used 5-point Likert scales. Two questions were exclusive to inpatient physicians as the authors of the diagnosis section (questions 5 and 11); all other questions were asked of both groups to allow comparison. Section (3) assessed content preferences based on a list of 36 potential content elements derived from the literature review and further validated and expanded through the FGD. Physicians rated each content element as “mandatory (shall),” “required, if available (should),” “desirable, if available (may),” or “not necessary,” following the conformance indicators used in HL7 CDA and those used in Germany’s forthcoming digital discharge summary (“Krankenhaus-Entlassbrief”).^48^ Consensus was defined as > 75% agreement, and strong consensus as > 95%, per Commission of the Association of Scientific Medical Societies (AWMF) guidelines.^49^ Section (4) assessed structural preferences using a stated-choice experiment, where physicians selected their preferred format for each of seven structure-related questions, each with pre-tested visual examples. Section (5) asked single-choice questions on demographics. See Supplementary Note 3 for a translated version of the survey questionnaire.

The survey also included a free-text question asking physicians what additional features they would want in an ideal digital diagnosis section beyond what was covered in the survey. These comments were qualitatively analyzed following Mayring’s eight steps of qualitative content analysis.^50^ The analysis was conducted using MAXQDA (version 24.4).^43^

**Figure 2:**
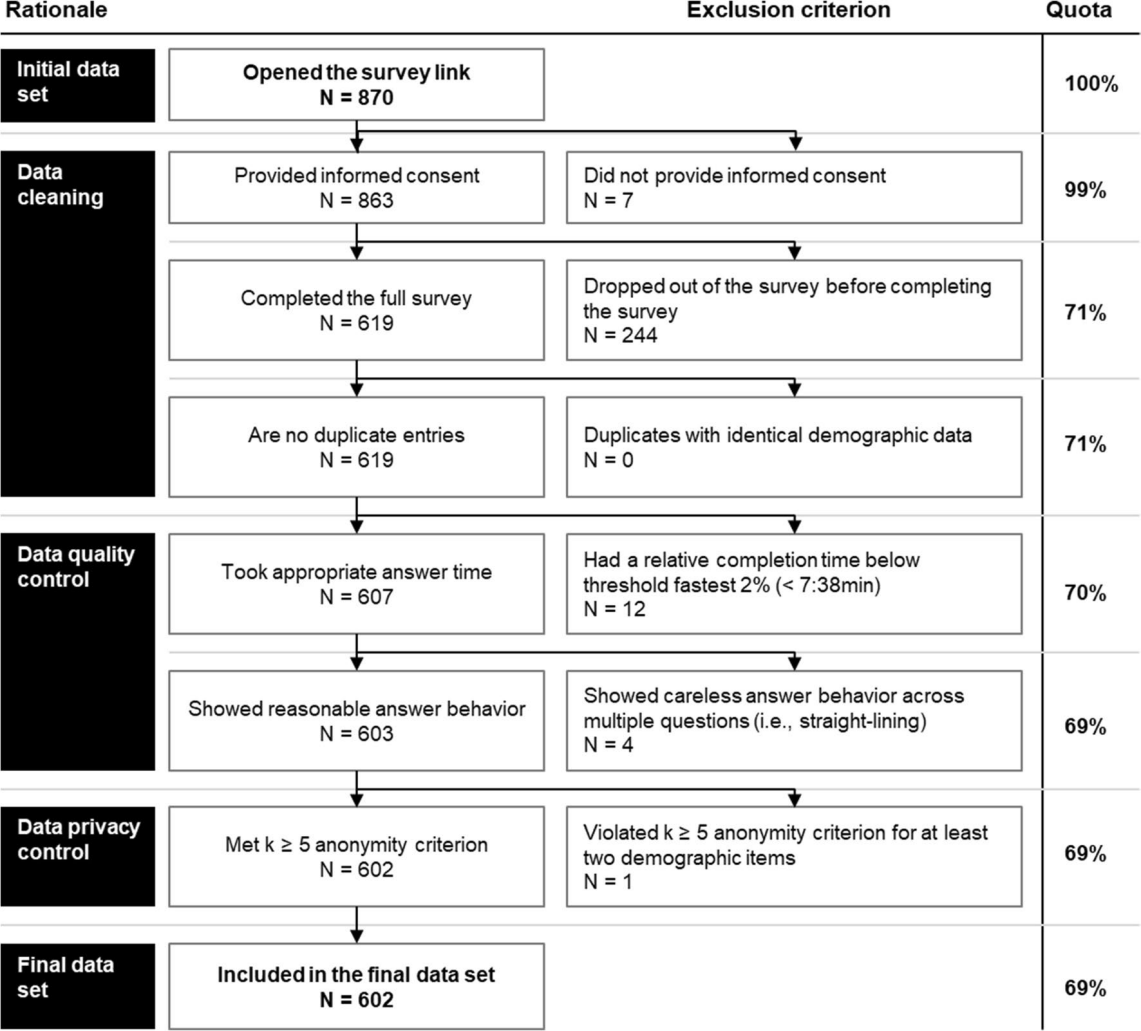
This figure shows the sequential data cleaning approach, detailing the rationale, exclusion criteria, and the number of remaining responses at each step (Quota). “*N*” represents the number of respondents excluded or advanced to the next stage of data cleaning.

Data cleaning followed standard best practices and is presented in Fig. 2.^51,52^ Descriptive statistics were calculated and differences between inpatient and outpatient physicians were assessed using the chi-square test for categorical variables and the Mann–Whitney *U* test for Likert scale data, with a significance level of *α* = 0.05. All statistical analyses were conducted using R Statistical Software (version 4.2.1).^53^

## RESULTS

### Literature Review

The initial search yielded 2376 articles, with 595 duplicates (see Fig. 1). After screening titles and abstracts, reviewing full texts in accordance with the inclusion criteria, and critical appraisal, 26 articles were selected for final review. Supplementary Table 7 provides the extracted data from these studies.

Nearly all (23) emphasized the importance of a clear and accurate diagnosis section, with some highlighting it as the most critical section of discharge summaries.^32,54–57^ Nine articles proposed some form of template, all of them disease- or specialty-specific.

#### Content of the Diagnosis Section

The following content elements were mentioned in multiple reports as key content elements in the diagnosis section: ICD-10 code,^58–60^ severity/stage classification,^60,61^ disease course and duration for chronic diseases,^62^ initial diagnosis date,^63^ comorbidities,^64^ complications,^64,65^ and any associated surgeries or interventions including dates.^64–66^ Cardiovascular risk factors,^67^ allergies,^65^ and tobacco or alcohol abuse^65^ should also be included. Acronyms and jargon should be avoided when detailing diagnoses.^32,60,62^ A common theme was that outpatient physicians want clear guidance on required follow-up actions along with each diagnosis, which inpatient physicians often omit.^59,68^

The review also revealed that physicians prefer detailed, disease-specific information for each diagnosis. For example, for (pre-)diabetic patients, including the admission and target HbA1c with the diagnosis was considered important.^67^ In cases involving blood transfusions, key details such as the date, number of transfusions, occurrence of reactions, and reason for the transfusion were seen as important.^69^ For permanent pacemaker insertions, physicians expected information on the date, indication, pacemaker type, make and model, access route, and complications.^70^ One review therefore suggested using diagnosis-specific templates to help clinicians include pertinent information.^30^ Another review suggested a three-point diagnosis checklist to ensure completeness and accuracy, clear structure, and relevance.^71^

#### Structure of the Diagnosis Section

The literature revealed that physicians prefer a highly structured format^56^ ideally with a standardized layout across medical specialties.^12,54,71^ Diagnoses should be arranged by clinical relevance,^55,66^ with causally related diagnoses grouped together.^60–62,66^ Some reports suggested listing relevant surgeries alongside diagnoses,^62,66^ while others recommended placing surgeries under a separate heading.^60,61^ Similarly, there was no consensus on how to organize diagnoses with headings: four reports advocated separating current treated diagnoses from past or additional diagnoses,^60,65,67,72^ while three studies suggested dividing diagnoses into primary and secondary diagnoses, often for hospital billing or coding purposes.^66,73,74^

### Focus Group Discussion

Experts in the FGD unanimously stressed the diagnosis section’s critical role in transmitting transition-of-care information, with one physician remarking, “Outpatient physicians only read the diagnosis section, medication plan, and summary; there is no time for more.” They highlighted the poor quality of many diagnosis sections, citing factors such as the lack of structural and content standards, time constraints, and limited German proficiency among some junior physicians. The discussion generated a list of potential reasons for inadequate diagnosis sections, which informed the survey answer choices.

### Online Survey

After removing 251 responses during data cleaning, 16 during quality checks, and 1 due to privacy controls (see Fig. 2), 602 responses (317 outpatient and 285 inpatient physicians) were included in the analysis. Respondent characteristics are summarized in Table 1, reflecting a broad cross-section of the German physician population.

**Table 1:**
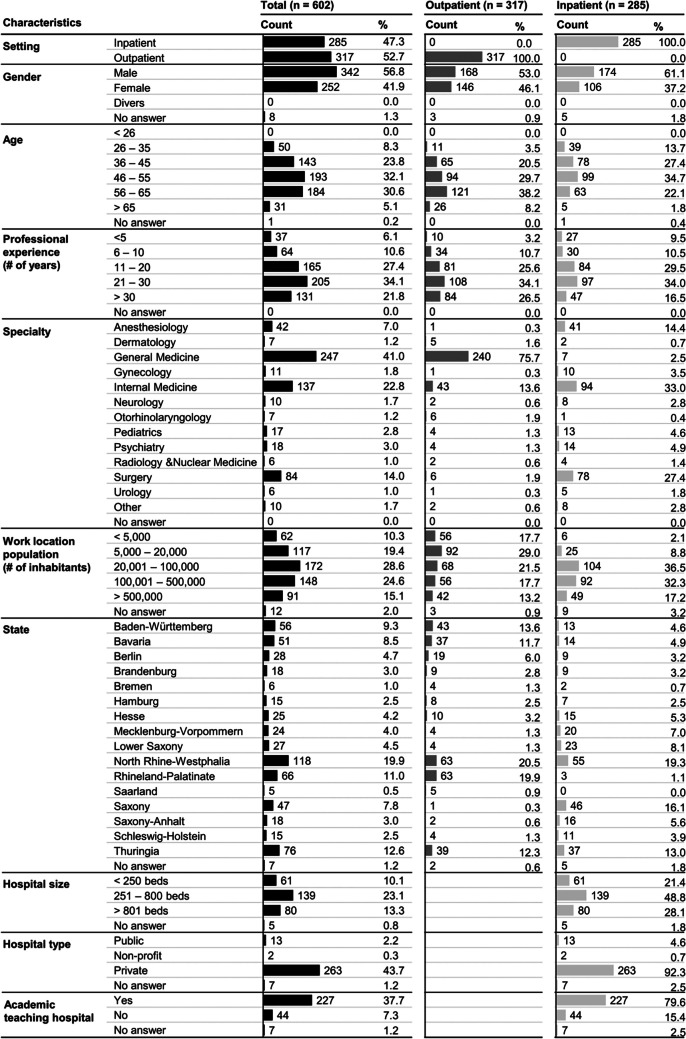
Characteristics of Physician Survey Sample (*n* = 602).

#### Usage, Importance of, and Satisfaction with the Diagnosis Section

The diagnosis section serves multiple purposes: 88.2% of physicians reported using it to gain a brief overview of the current illness, 65.0% to transfer diagnoses into their IT systems, and 55.5% to obtain a comprehensive picture of the patient. Inpatient physicians (67.0%) were more likely than outpatient physicians (45.4%) to use it for a comprehensive view of the patient (*p* < 0.001), while outpatient physicians (79.8%) were more likely than inpatient physicians (48.2%) to use it for transferring diagnoses into their IT systems (*p* < 0.001) (see Supplementary Fig. 1).

Nearly all physicians (95.7%) agreed or strongly agreed on the importance of the diagnosis section for continued patient treatment (*M* = 4.62, SD = 0.77). However, satisfaction with the diagnosis section varied widely. Among physicians, 35.9% were unsatisfied and 27.2% were neutral. On average, physicians reported being fully satisfied with approximately half of the diagnosis sections they receive. Considering this variability in satisfaction, nearly all physicians (91.2%) agreed or fully agreed that “structural and content standards in the diagnosis section would facilitate the accurate and unambiguous transmission of treatment-related information.”

#### Reasons for Insufficient Diagnosis Sections

Ten underlying reasons were rated by over 50.0% of inpatient physicians as very relevant or relevant contributors to insufficient diagnosis sections (see Supplementary Fig. 2). The most critical factors cited were “Copy-pasting from prior discharge summaries without review” (96.1%), “time pressure” (89.4%), “deficient admission documentation” (82.6%), and “lack of general guidelines/conventions” (70.4%).

#### Audiences of the Diagnosis Section

Inpatient physicians rated capturing information in the diagnosis section as most important for “physicians providing follow-up care” (96.8% important or very important), followed by “colleagues in the clinic” (95.0%) and “myself” (94.3%). It was also considered important for other recipients such as “medical coders” (73.3%) and “patients” (70.1%). The lowest importance was assigned to capturing information for “health insurance” (62.6%).

#### Importance of and Satisfaction with Subsections of the Diagnosis Section

**Figure 3:**
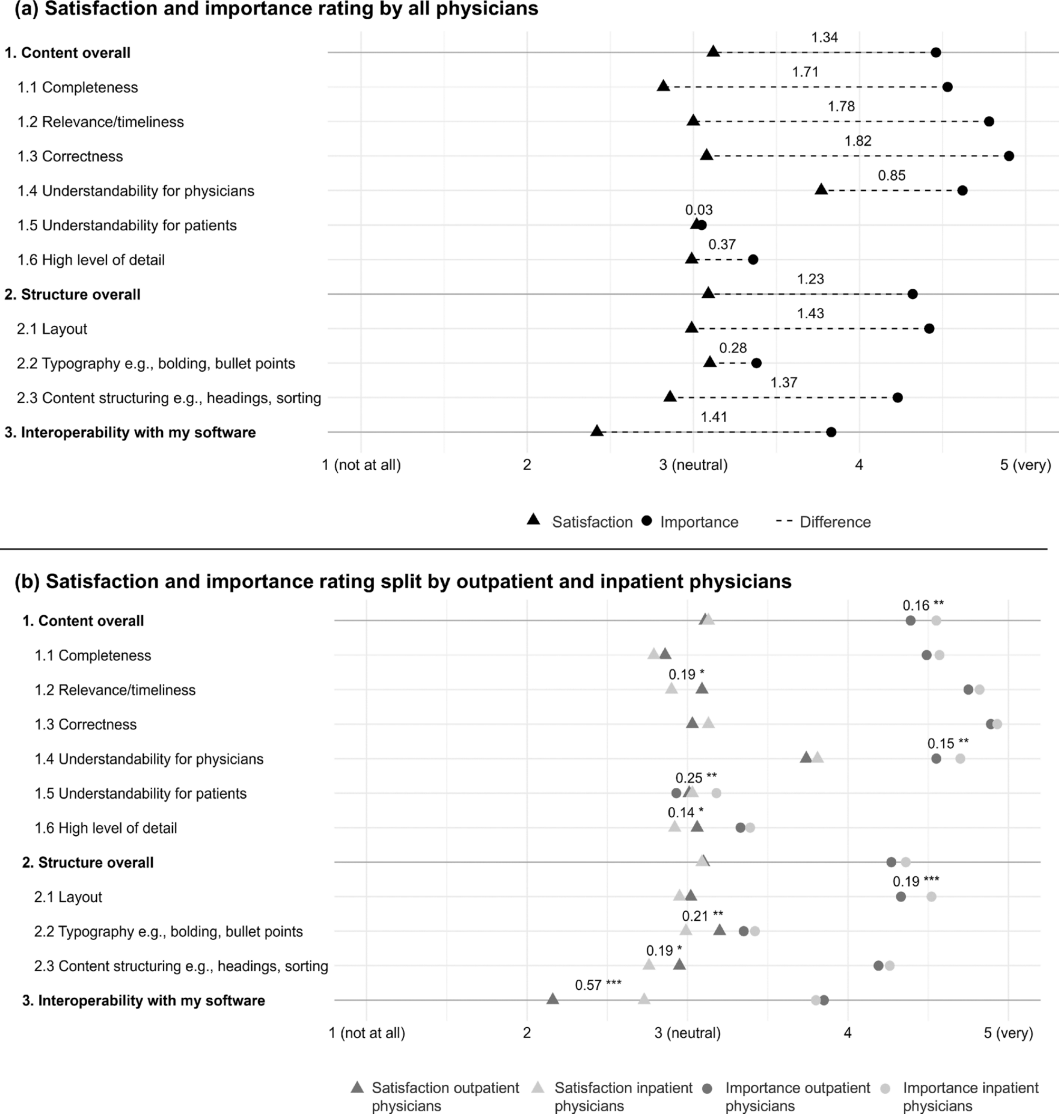
This figure presents physicians’ ratings of importance and satisfaction with the diagnosis section on a 5-point Likert scale across three areas: (1) overall content and six sub-elements, (2) overall structure and three sub-elements, and (3) interoperability with hospital/practice software. The triangle indicates mean satisfaction, while the circle represents mean importance. Panel **a** displays the mean importance and satisfaction ratings for the full sample (*N* = 602), with the difference between them visualized with a dotted line. Panel **b** compares the ratings between outpatient physicians (dark gray) and inpatient physicians (light gray). Statistically significant differences between the two groups were assessed using the Mann–Whitney *U* test, with significance levels denoted as follows: *p < 0.05, **p < 0.01, ***p < 0.001.

Figure 3 illustrates physicians’ ratings of the importance and satisfaction with various aspects of the diagnosis section. “Content” was rated as the most important (*M* = 4.46, SD = 0.72), followed by “structure” (*M* = 4.32, SD = 0.67) and “interoperability with software” (*M* = 3.83, SD = 1.1). Among content aspects, “correctness” is considered the most important factor (*M* = 4.90, SD = 0.37), followed by “relevance/timeliness” (*M* = 4.78, SD = 0.49) and “completeness” (*M* = 4.53, SD = 0.74). In contrast, “understandability for patients” (*M* = 3.05, SD = 1.01) was rated as the least important. For structure, “layout,” i.e., the overall visual arrangement and design of the diagnosis section like paragraph format or tabular structure (*M* = 4.42, SD = 0.67) and “content structuring, e.g., headings, sorting” (*M* = 4.23, SD = 0.77) were rated as important.

Physicians expressed general dissatisfaction with most aspects of the diagnosis section, especially when comparing satisfaction to the perceived importance, with a difference in means greater than one for most categories. Exceptions were seen for “understandability for patients,” where the difference in means was only 0.03. Physicians were most satisfied with “understandability for physicians” (*M* = 3.77, SD = 0.74), with 74% reporting satisfaction. Physicians were least satisfied with “interoperability with software” (*M* = 2.42, SD = 1.01) and “completeness” (*M* = 2.82, SD = 0.91). The largest gaps between importance and satisfaction were observed for “correctness” (difference, 1.82) and “relevance/timeliness” (difference, 1.78).

Outpatient physicians reported significantly lower satisfaction with the interoperability of the diagnosis section with their practice software, with only 7.6% expressing satisfaction compared to 15.0% of inpatient physicians (*p* < 0.001). Additionally, significant differences between physician groups were found in satisfaction with “typography, e.g., bolding, bullet points” (*p* = 0.002) and in the perceived importance of “overall content” (*p* = 0.004), “understandability for patients” (*p* = 0.003), “understandability for physicians” (*p* = 0.005), and “layout” (*p* < 0.001) (see Fig. 3b).

#### Content Elements Preferred in the Diagnosis Section

**Figure 4:**
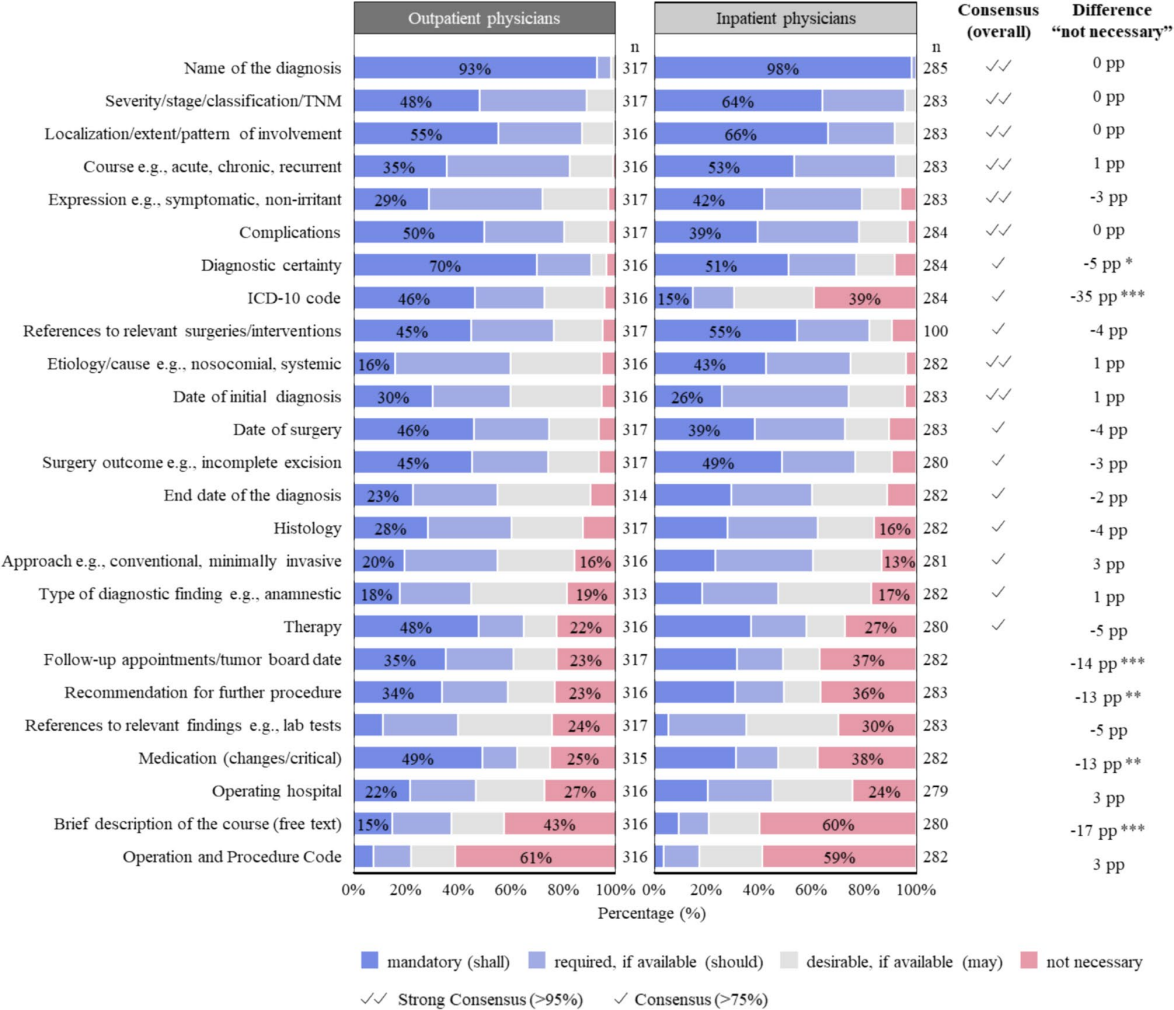
This figure shows the ratings by outpatient and inpatient physicians on the inclusion of 25 content elements in the diagnosis section. Each content element was rated as “mandatory (shall),” “required, if available (should),” “desirable, if available (may),” or “not necessary,” following the conformance indicators used in CDA and Germany’s upcoming digital discharge summary (“Krankenhaus-Entlassbrief”). “*n*” represents the number of responses for each content element from inpatient and outpatient physicians. Consensus for inclusion is marked with a double checkmark for strong consensus (> 95% agreement) and a single checkmark for consensus (> 75% agreement), in line with AMWF guidelines. Agreement includes all ratings except “not necessary.” Differences in the “not necessary” ratings between inpatient and outpatient physicians are shown in percentage points (pp). Statistically significant differences were evaluated using the chi-square test, with significance levels indicated as follows: *p < 0.05, **p < 0.01, ***p < 0.001.

Figure 4 presents the ratings of all content elements, split by inpatient and outpatient physicians. Eighteen out of 25 content elements are rated as “mandatory (shall),” “required, if available (should),” or “desirable (may)” by more than 75.0% of all physicians, meeting the consensus threshold set by the AWMF. This indicates broad agreement on the inclusion of these elements for each active treatment diagnosis. Among outpatient physicians alone, 21 of the 25 content elements reached the 75.0% consensus for inclusion.

The content elements deemed most important, achieving strong consensus (> 95.0% of physicians rating them as “mandatory,” “required,” or “desirable”), are as follows: “name of the diagnosis” (100.0%), “severity/stage/classification/TNM” (99.8%), “localization/extent/pattern of involvement” (99.5%), “course e.g., acute, chronic, recurrent” (99.3%), “expression, e.g., symptomatic, non-irritant” (97.0%), “complications” (95.7%), “date of initial diagnosis” (95.3%), and “etiology/cause (e.g., nosocomial, systemic)” (95.2%). In contrast, the content elements rated as least necessary are “operations and procedure code” (59.9% rated as “not necessary”) and “brief description of the course” (50.7% rated as “not necessary”).

Inpatient and outpatient physicians disagreed over the perceived necessity of including “ICD-10 code”: among outpatient physicians, 95.6% rated “ICD-10 code” as either “mandatory” (46.2%), “required” (26.5%), or “desirable” (22.8%), compared to only 14.8% of inpatient physicians rating it as “mandatory.” Inpatient physicians were also significantly more likely (*p* < 0.01) than outpatient physicians to rate the following elements as “not necessary”: “follow-up appointments/tumor board date” (14 percentage points higher) and “recommendations for further procedure” (13 percentage points higher).

Regarding surgery-related content elements, “references to relevant surgeries” achieved a 93.0% consensus, whereby including “complications” (95.7%), “date of surgery” (92.1%), and “surgery outcome” (91.5%) was rated as particularly important. In contrast, mentioning the “operating hospital” was considered less critical (74.1%).

Beyond content elements related to the current treatment diagnosis, physicians also preferred including additional elements in the diagnosis section, with the following reaching a consensus rating for inclusion of over 75%: “Infection/colonization by multidrug-resistant bacteria” (97.3%), “allergies/intolerances” (94.0%), “cardiovascular risk factors” (87.4%), “substance use/toxins” (84.7%), “implants and medical devices” (82.0%), and “pending investigations/findings” (75.0%). See Supplementary Fig. 3.

#### Use of Abbreviations

Only 26.8% of physicians agreed or strongly agreed that using abbreviations increased efficiency in reading and writing the diagnosis section. Over half of physicians, 55.9%, disagreed or strongly disagreed that the potential time and space savings from using abbreviations outweigh the risk of miscommunication. A total of 71.1% of physicians agreed that no specialty-specific abbreviations should be used in the diagnosis section.

#### Format and Structure Preferred for the Diagnosis Section

The stated preference experiment revealed that physicians favored either a paragraph (52.4%) or tabular (45.9%) format for the diagnosis section. Additionally, 42.2% preferred highlighting all important information, while 29.0% favored highlighting only current treatment diagnoses. Physicians preferred listing diagnoses without numbering (40.9%) or numbering only the main diagnoses (31.3%). Nearly all physicians (93.2%) agreed that diagnoses should be sorted by clinical relevance.

Most physicians (94.6%) supported some form of content structuring (e.g., headings), with 44.9% preferring to separate “current treatment diagnoses” from “chronic and prior diagnoses” under a heading, while 41.5% supported also separating “chronic diagnoses” from “prior diagnoses.” Regarding surgical procedures, 49.4% favored listing surgeries alongside diagnoses, while 30% preferred a separate heading for surgical procedures. See Supplementary Table 8.

#### Interactive Content Elements

A significant majority of physicians (83.1%) expressed a preference for including interactive elements in a digital diagnosis section. The most requested features were “links (e.g., to findings)” (59.8%), “expandable elements (e.g., to show and hide details for diagnoses)” (46.9%), and a “search function” (33.9%). Outpatient physicians showed a stronger preference for a “filter function” compared to inpatient physicians (30.9% vs. 25.3%), while inpatient physicians favored “links” more than outpatient physicians (62.5% vs. 57%). See Supplementary Fig. 4.

#### Qualitative Analysis of Free-Text Comments

One hundred thirty-five free-text comments were received. Of these, 40 comments stressed the urgent need for a uniform, structured diagnosis section. One physician noted: “Each hospital and doctor uses their own layout. This lack of uniformity leads to diagnoses and ‘to-do’s’ being overlooked.” Another highlighted the importance of structure for quickly understanding inpatient stays: “Ideally, everything should be visible at a glance.” Others echoed this, noting that a well-structured section reduces the risk of overlooking information and may prevent treatment errors.

Fifteen comments identified key pitfalls for a digital diagnosis section, notably the need to avoid excessive documentation: “We must avoid excessive documentation effort to maintain a digital diagnosis section.” Others expressed concerns about information overload: “Do not overload with data clutter” and “excessive information can turn the diagnosis section into a lengthy medical history, filled with redundant content.” Eleven comments echoed the need for succinctness, urging that the diagnosis section remain concise and focused.

Fourteen comments from outpatient physicians reiterated the necessity of including ICD-10 codes, noting the time savings during data entry: “The time savings would be incredible (…) we’re talking about approx. 30–45 min. per day!!” Twelve comments advocated for including next steps and treatment recommendations within the diagnosis section: “For a GP, it is important to quickly understand why the patient was there and what needs to be done afterward, e.g., continuation of antibiotics.” Another added, “If something should be checked after 3 months – that absolutely needs to be mentioned with the diagnosis.” Outpatient physicians also stressed the need to include “upcoming appointments for follow-up treatment” alongside the diagnoses.

Several comments requested support for abbreviations, suggesting features like hover-over text to display their full meaning. Outpatient physicians reported frequently needing to look up abbreviations, especially those from surgery, orthopedics, and ophthalmology.

No additional content elements for the diagnosis section were suggested beyond those already covered in the quantitative survey.

## DISCUSSION

### Implications of Findings: The Need for a Uniform Cross-Specialty Diagnosis Section

In healthcare systems like Germany’s, where care is divided between inpatient and outpatient settings, effective and efficient transfer of information via discharge summaries is crucial for continuity of care and patient safety. A complete and accurate diagnosis section is paramount, with 95.7% of physicians in this study confirming its importance for subsequent care, consistent with previous research.^32,54–57^

Despite its importance, only 36.9% of physicians expressed satisfaction with the diagnosis section overall, with even lower satisfaction reported for aspects such as completeness, content structuring (e.g., headings) and interoperability, corroborating prior research.^75,76^ Qualitative insights further revealed a perceived decline in diagnosis section quality over time, answering RQ1: while deemed essential, the diagnosis section often falls short of physician expectations.

Outpatient physicians, in particular, expressed dissatisfaction with the lack of interoperability. Concerns were also raised about the lack of uniformity, with hospitals, specialties, and individual doctors using different layouts, increasing the risk of important diagnosis information being overlooked. This highlights the need for an interoperable diagnosis section, achievable with cross-specialty standards for structure and content. The desire for such standardization is evident; 91.2% in this study agreed that standardized structural and content elements would facilitate the accurate and unambiguous transmission of treatment-related information, addressing RQ2.

A uniform digital diagnosis section must address key underlying issues for unsatisfactory diagnosis sections identified in this study such as copy-pasting from prior discharge summaries without review, time pressure, and the absence of clear guidelines, all of which are associated with higher readmission rates.^14,77^ A cross-specialty standard for structure and content would provide essential guidelines, likely reducing copy-paste errors by replacing free-text fields with standardized entries, thereby improving consistency and quality. Time pressure could also be alleviated by using a standardized template that auto-suggests content elements or incorporate generative AI language models,^78,79^ streamlining the documentation process. However, standardization alone is insufficient. Complementary strategies, such as education,^80–82^ ongoing feedback,^83,84^ (financial) incentives for high-quality documentation,^85,86^ and reduced physician workload,^87^ are also needed to enhance the quality of discharge documentation.

### Proposed Cross-Specialty Discharge Summary Standard Based on Physician Preference

As noted in the literature review, previous studies have proposed discharge templates tailored to specific specialties or diseases, but these are typically implemented only within individual departments or hospitals. Our findings indicate that a standardized diagnosis section across all specialties is essential, especially for outpatient physicians who receive them from multiple hospitals, each with its own layout. While disease-specific templates can define the specific required, recommended, or optional content elements for specific diagnoses, the overall structure should remain consistent across specialties.

**Figure 5:**
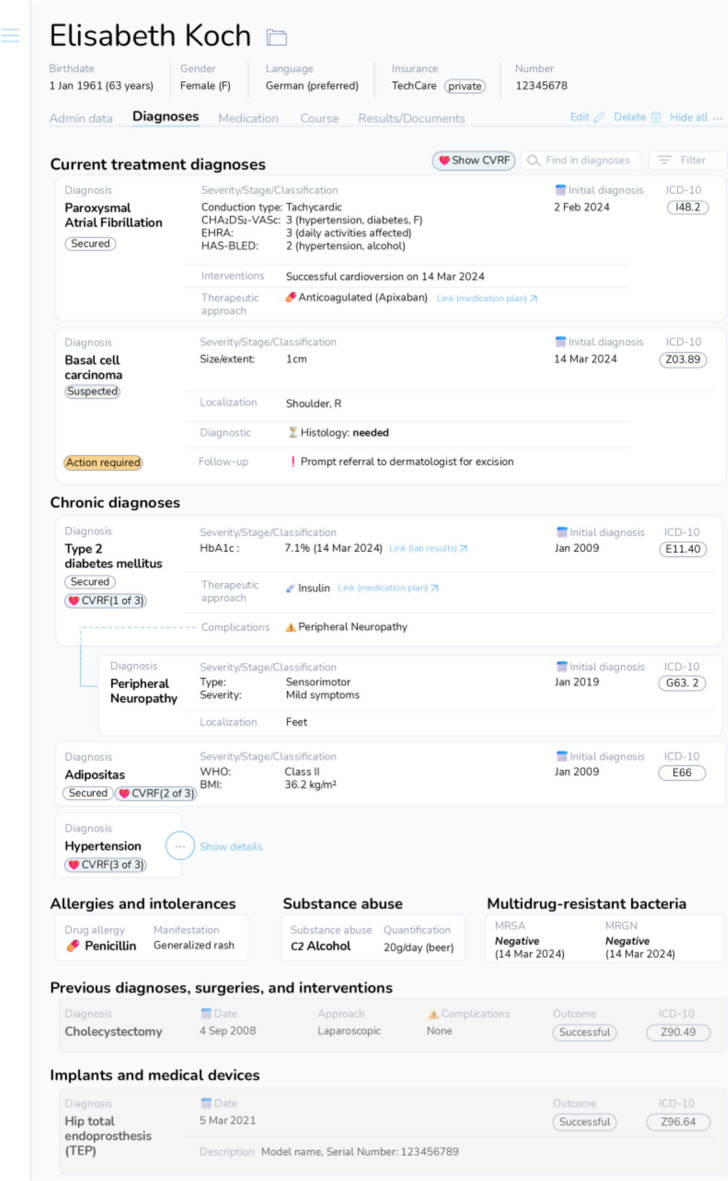
This figure presents a visualization of a potential digital diagnosis section, designed according to the consensus preferences of German physicians in this study (*N* = 602). The structure reflects physician preferences, with content elements that achieved (strong) consensus provided for current treatment, chronic, and previous diagnoses. Diagnoses are automatically encoded using SNOMED-CT and ICD-10 codes. Upon entering an ICD-10 code, the template automatically populates the relevant fields for the diagnosis. Diagnoses treated during the current hospital stay are separated from chronic, active conditions and clinically inactive, previous diagnoses, surgeries, and interventions. Additionally, allergies, substance abuse, implants and medical devices, and infection/colonization with multi-drug-resistant bacteria are listed under separate headings. Critical follow-up steps are highlighted and color-coded for physicians providing follow-up care. The digital diagnosis section features interactive elements, such as links to further details (e.g., medication plan, lab test results) and search/filter functionality (e.g., displaying all cardiovascular risk factors with one click). The data is fully interoperable with electronic patient records and can be seamlessly transferred to subsequent care providers. Additionally, customizable views allow users to show or hide information based on their preferences or needs.

In addressing RQ3, our study identified the content and structural elements that German physicians prefer for the diagnosis section. Based on the elements that achieved consensus, a potential visualization for a uniform digital diagnosis section is shown in Fig. 5. Additionally, our findings highlighted that patients and medical coders, alongside physicians providing follow-up care, are key audiences for the diagnosis section. Documentation system design must accommodate these diverse users, potentially through customizable viewer profiles that adjust the content or level of detail based on the audience.

### Different Views on the Diagnosis Section Between Inpatient and Outpatient Physicians

In addressing RQ4, this study revealed that the diagnosis section served different purposes for inpatient and outpatient physicians, and that their views on how comprehensive it should be often diverged. Outpatient physicians generally preferred a more detailed diagnosis section, placing greater importance on elements such as ICD-10 codes, follow-up appointments, treatment recommendations, and free-text descriptions of the patient’s course compared to inpatient physicians.

These preferences likely reflect the different ways the two groups use the diagnosis section. Outpatient physicians often need to transfer diagnoses into their IT systems (79.8% of outpatient vs. 48.2% of inpatient physicians), explaining their focus on ICD-10 codes. Conversely, inpatient physicians rely more on the diagnosis section to gain a comprehensive picture of the patient (67.0% vs. 45.4%). Outpatient physicians, typically familiar with the patient’s history, prioritize recent hospital events and future care, while inpatient physicians, often seeing the patient for the first time, need an overall understanding, including past diagnoses. These divergent preferences mirror findings from surveys in the USA,^88^ the UK,^32,57^ and Austria.^54^

The proposed template helps reconcile these differences by offering a structured format that addresses both groups’ needs. While consensus was reached on 18 content elements, both qualitative feedback and existing literature emphasize the importance of brevity to avoid overwhelming users.^12,32,55,71^ The challenge of balancing comprehensiveness with conciseness is further exacerbated by the growing complexity of hospitalizations and required post-discharge care, particularly for geriatric patients with multiple comorbidities. This makes a structured, easy-to-read format even more critical.

### Practical Recommendations for Writing Diagnosis Sections

Based on the consensus findings of this study, Table 2 offers practical recommendations for writing diagnosis sections — a daily and often challenging task for junior doctors in hospitals worldwide.^85,89^

**Table 2:** Practical Advice for Writing the Diagnosis Section of Discharge Summaries.

Category	Recommendation
General guidelines	• *Purpose*: Aim to effectively transfer vital diagnostic information to physicians providing follow-up care • *Audience*: Ensure it is targeted and understandable for colleagues from different specialties and especially primary care physicians • *Brevity*: Keep it as concise as possible while including all relevant information • *Relevance*: Include only the most pertinent information; assume the entire discharge summary may not be read • *Clarity*: Use clear, precise, and easy-to-read language • *No abbreviations*: Spell out terms to ensure better readability • *Standard terminology*: Use SNOMED-CT terminology and include ICD-10 codes for every diagnosis • *Alignment*: Ensure all recommendations, treatments, and follow-up actions correspond to the relevant diagnosis • *Consistency*: Check that all medications in the medication plan have a corresponding diagnosis in the diagnosis section
Structure to follow	• *Layout*: Use a structured layout, ideally following the template proposed in this study • *Headings*: Separate current treatment diagnoses from relevant previous/chronic diagnoses • *Order*: List current diagnoses by clinical importance, grouped by etiology/cause, where applicable • *Additional sections*: Include specific sections (e.g., surgical/oncological course, allergies) as needed
Content to include	• *Diagnoses*: List diagnoses, not symptoms • *Details*: Include at minimum stage/severity/classification/TNM, date of initial diagnosis, and as relevant localization/extent/pattern of involvement, course, expression, complications, and etiology/cause • *Follow-up actions*: Clearly indicate any required actions by the follow-up physician, alongside the relevant diagnosis • *No irrelevant entries*: Remove duplicate diagnoses and consolidate related diagnoses to maintain clarity

### Limitations and Future Research

Several limitations must be acknowledged. First, there is potential for nonresponse bias, though we sought to minimize this by ensuring a broad and diverse sample. Second, sampling bias may exist due to our use of the Helios physician mailing list for inpatient physician recruitment. While Helios covers 87 hospitals of varying sizes and specializations, the findings may not fully represent physicians in public or non-profit hospitals. Additionally, focusing solely on physicians as the primary authors and readers of the diagnosis section, may limit the generalizability of our findings to other users, such as nurses, clinical coders, and patients.

Despite these limitations, this study is the largest to date investigating physician preferences regarding diagnosis documentation and is the first to examine the diagnosis section in such detail.

Future research should expand on this study by including the perspectives of other users of the diagnosis section and physicians from different countries. The next step is to develop diagnosis-specific templates that detail the precise disease-specific information that shall, should, or may be included for each content element. This will be undertaken by the authors as part of a larger research project.

Once these templates are created in the standardized format, they must be tested in real-world settings to assess their impact on documentation accuracy, physician satisfaction, and, most importantly, patient outcomes. After validation, these structural and content standards should be integrated into future documentation systems, including Germany’s digital discharge summary (“KH-Entlassbrief”).

## CONCLUSION

This study aimed to determine a cross-specialty standard for the structure and content of a digital diagnosis section in hospital discharge summaries by capturing the preferences of German physicians. Using a comprehensive mixed-methods approach, we explored key aspects of the diagnosis section’s importance, physician satisfaction, and content and structural preferences, culminating in a proposed format. The proposed format could serve as a future standard, comparable to the standardized medication plan in Germany (“Bundeseinheitlicher Medikationsplan”). The findings also reaffirmed the importance of involving physicians early in the design of digital documentation systems to ensure alignment with clinical workflows and user needs, ultimately enhancing patient care and outcomes.

## Supplementary Information

Below is the link to the electronic supplementary material.

Supplementary file1 (PDF 1.54 MB)

## Data Availability

The datasets used and/or analyzed during the current study are available from the corresponding author on reasonable request.
