# Supplementary material for: Diagnosis Documentation Done Right: Cross-Specialty Standard for the Diagnosis Section in German Discharge Summaries — A Mixed-Methods Study
Source: J Gen Intern Med. 2025 Feb 6;40(6):1387–402. doi: 10.1007/s11606-025-09395-9 (PMC12045923; doi:10.1007/s11606-025-09395-9)
Supplement: Supplementary file 1 — Supplementary file1 (PDF 1.54 MB) [file 11606_2025_9395_MOESM1_ESM.pdf]

Supplementary Table 1 PRISMA extension for scoping reviews (PRISMA-ScR) checklist

SECTION	ITEM	PRISMA-ScR CHECKLIST ITEM	REPORTED IN SECTION
**TITLE**			
Title	1	Identify the report as a scoping review.	N/A (in abstract)
**ABSTRACT**			
Structured summary	2	Provide a structured summary that includes (as applicable): background, objectives, eligibility criteria, sources of evidence, charting methods, results, and conclusions that relate to the review questions and objectives.	Background and Objectives: The diagnosis section of hospital discharge summaries plays a critical role in patient care transitions. However, variations in its content and structure create challenges for both inpatient and outpatient physicians. This review aimed to synthesize existing literature on physician preferences for content and structure in the diagnosis section and inform the development of a uniform standard.<br>Methods: A systematic scoping review was conducted following PRISMA-ScR guidelines, searching PubMed, Scopus, Cochrane, and MEDLINE databases, with additional snowball searches. A total of 2,376 articles were screened, with 26 meeting the inclusion criteria after critical appraisal. Data were extracted and key themes from the literature synthesized.<br>Findings: Physicians prefer detailed, disease-specific information in the diagnosis section, with key elements including ICD-10 codes, comorbidities, complications, and follow-up actions. A clear, structured format was emphasized, with conflicting views on whether surgeries should be listed with diagnoses or under separate headings. Standardized, interdisciplinary templates were recommended to improve clarity and reduce variability.<br>Interpretation: This review highlights the need for a uniform, structured diagnosis section that incorporates detailed, relevant information. Developing standardized templates based on physician preferences can enhance discharge summary quality, reduce information overload, and improve patient care continuity.
**INTRODUCTION**			
Rationale	3	Describe the rationale for the review in the context of what is already known. Explain why the review	Extensive research on discharge summaries exists, but much of it is limited to audits of their content. This review aims to provide a comprehensive overview of

SECTION	ITEM	PRISMA-ScR CHECKLIST ITEM	REPORTED IN SECTION
		questions/objectives lend themselves to a scoping review approach.	studies on physicians' preferences regarding the structure and content of discharge summaries, with a specific focus on the diagnosis section. While the majority of research has examined discharge summaries as a whole, detailed investigations into preferences for the diagnosis section—one of the most critical components—remain limited. This review seeks to identify and assess the existing scope of research on physicians' preferences in discharge documentation.
Objectives	4	Provide an explicit statement of the questions and objectives being addressed with reference to their key elements (e.g., population or participants, concepts, and context) or other relevant key elements used to conceptualize the review questions and/or objectives.	The objective of the review was to gain an overview and understanding of the current scope of research on discharge summary documentation preferences of physicians, and particularly any insights on preferences for the diagnosis section. The aim was to inform the FGD and questionnaire design. The following research questions (RQ) were addressed: How important is the diagnosis section within discharge summaries to physicians, and how satisfied are they with its current state? Do physicians support the establishment of structural and content standards for discharge summaries? What content and structural elements do physicians prefer for the discharge summary, specifically the diagnosis section? How do preferences for content and structure differ between inpatient and outpatient physicians?
**METHODS**			
Protocol and registration	5	Indicate whether a review protocol exists; state if and where it can be accessed (e.g., a Web address); and if available, provide registration information, including the registration number.	As this review was part of a mixed-methods approach, we did not prepare, register, nor publish a review protocol.
Eligibility criteria	6	Specify characteristics of the sources of evidence used as eligibility criteria (e.g., years considered, language, and publication status), and provide a rationale.	Five inclusion criteria were predetermined to select relevant research. The research must: (1) look at written discharge communication (as opposed to discharge processes in general), (2) look at discharge communication from inpatient physicians to outpatient physicians (as opposed to within or between hospitals or from nurses or to pharmacists), (3) cover physician's preference for content or structure of the discharge summary (as opposed to mere audits of discharge

SECTION	ITEM	PRISMA-ScR CHECKLIST ITEM	REPORTED IN SECTION
			summaries), (4) be written in English or German, and (5) be published in a medium accessible online. No date restrictions were applied.
Information sources	7	Describe all information sources in the search (e.g., databases with dates of coverage and contact with authors to identify additional sources), as well as the date the most recent search was executed.	We searched the PubMed, Scopus, Cochrane, and MEDLINE databases. We also searched for guidelines and other articles using Google as well as hand-searched bibliographies (snowball method).
Search	8	Present the full electronic search strategy for at least 1 database, including any limits used, such that it could be repeated.	To identify potentially relevant articles, we developed a search strategy using the Boolean OR operator to combine 14 terms related to discharge communication and 18 terms covering content, structure, and levels of detail preferences. This search was applied to titles and abstracts, with Medical Subject Headings included for PubMed.<br><br>PubMed search string for abstract and title: ((Discharge letter*[Title/Abstract]) OR (Discharge summar*[Title/Abstract]) OR (Discharge document*[Title/Abstract]) OR (Discharge paper*[Title/Abstract]) OR (Doctor* letter*[Title/Abstract]) OR (Physician* letter*[Title/Abstract]) OR (Doctor-Doctor[Title/Abstract]) OR (Physician-Physician[Title/Abstract]) OR (Arztbrief*[Title/Abstract]) OR (Entlassbrief*[Title/Abstract]) OR (Entlassungsbrief*[Title/Abstract]) OR (Krankenhaus-Entlassbrief*[Title/Abstract]) OR (Arzt-Arzt[Title/Abstract])) AND ((Content[Title/Abstract]) OR (Structure[Title/Abstract]) OR (Level of detail[Title/Abstract]) OR (Abbreviation*[Title/Abstract]) OR (Communication[Title/Abstract]) OR (Correspondence*[Title/Abstract]) OR (Satisfaction[Title/Abstract]) OR (Preference*[Title/Abstract]) OR (Quality[Title/Abstract]) OR (Pain Point*[Title/Abstract]) OR (Inhalt[Title/Abstract]) OR

SECTION	ITEM	PRISMA-ScR CHECKLIST ITEM	REPORTED IN SECTION
			(Struktur[Title/Abstract]) OR (Detaillierungsgrad[Title/Abstract]) OR (Kommunikation[Title/Abstract]) OR (Korrespondenz*[Title/Abstract]) OR (Zufriedenheit[Title/Abstract]) OR (Präferenz*[Title/Abstract]) OR (Qualität[Title/Abstract]))
Selection of sources of evidence	9	State the process for selecting sources of evidence (i.e., screening and eligibility) included in the scoping review.	See "Literature Review" section and PRISMA flow chart
Data charting process	10	Describe the methods of charting data from the included sources of evidence (e.g., calibrated forms or forms that have been tested by the team before their use, and whether data charting was done independently or in duplicate) and any processes for obtaining and confirming data from investigators.	We developed a data collection form in Excel to capture all relevant details from the included studies. The pre-formatted data collection form was discussed amongst all authors to ensure all variables are included to capture the necessary information.
Data items	11	List and define all variables for which data were sought and any assumptions and simplifications made.	We sought the following details: authors, country, year of publication, research aim, study design, specialty or disease area, outcomes, specific findings related to the diagnosis section, and whether a template was developed. The term template was interpreted broadly as the levels of detail varied greatly on what the authors understood as a template.
Critical appraisal of individual sources of evidence	12	If done, provide a rationale for conducting a critical appraisal of included sources of evidence; describe the methods used and how this information was used in any data synthesis (if appropriate).	The Joanna Briggs Institute (JBI) critical appraisal tools were chosen to assess the methodological quality of the selected reports (see Supplementary Table 4 for scores).
Synthesis of results	13	Describe the methods of handling and summarizing the data that were charted.	After extraction and charting of the data, the literature was synthesized into key themes, which were then discussed within the author group and subsequently in the FGD.
**RESULTS**			
Selection of sources of evidence	14	Give numbers of sources of evidence screened, assessed for eligibility, and included in the review, with reasons for exclusions at each stage, ideally using a flow diagram.	See "Literature Review" section and PRISMA flow chart
Characteristics of sources of evidence	15	For each source of evidence, present characteristics for which data were charted and provide the citations.	See "Literature Review" section, Fig. 1 and Supplementary Table 4
Critical appraisal within sources of evidence	16	If done, present data on critical appraisal of included sources of evidence (see item 12).	See Supplementary Table 4

SECTION	ITEM	PRISMA-ScR CHECKLIST ITEM	REPORTED IN SECTION
Results of individual sources of evidence	17	For each included source of evidence, present the relevant data that were charted that relate to the review questions and objectives.	See “Literature Review” section and Supplementary Table 7
Synthesis of results	18	Summarize and/or present the charting results as they relate to the review questions and objectives.	See “Literature Review” section and Supplementary Table 7
**DISCUSSION**			
Summary of evidence	19	Summarize the main results (including an overview of concepts, themes, and types of evidence available), link to the review questions and objectives, and consider the relevance to key groups.	See “Literature Review” section and Supplementary Table 7 summarizing the main results
Limitations	20	Discuss the limitations of the scoping review process.	First, although we used a search engine (Google) in addition to literature databases (PubMed, Scopus, Cochrane, MEDLINE) to identify relevant articles, it is possible that we did not capture all literature pertinent to our research question, and we may have encountered publication bias.<br><br>Second, as this review was part of a larger mixed-methods study, the search, data extraction, and synthesis were conducted by a single author (JF). However, we believe this did not affect the synthesis of results, as the findings were discussed among the authors and externally validated through a focus group discussion (FGD).
Conclusions	21	Provide a general interpretation of the results with respect to the review questions and objectives, as well as potential implications and/or next steps.	See “Literature Review”, “Results” and “Discussion” section
**FUNDING**			
Funding	22	Describe sources of funding for the included sources of evidence, as well as sources of funding for the scoping review. Describe the role of the funders of the scoping review.	This study was supported by the internal research fund of the Faculty of Health, School of Medicine, Witten/Herdecke University, Germany (Project IFF 2024-80). Julian Frings is supported by a doctoral scholarship from the German Academic Scholarship Foundation (“Studienstiftung des deutschen Volkes”).

Supplementary Note 1 Inclusion criteria for the literature review

The inclusion criteria for this review required that studies: (1) focus specifically on written discharge communication, excluding those addressing discharge processes more broadly; (2) examine communication between inpatient physicians and outpatient physicians, excluding studies related to intra-hospital communication, nurse communication, or communication with pharmacists; (3) explore physician preferences regarding the content or structure of discharge summaries, rather than audits or evaluations of discharge summaries; (4) be published in English or German; and (5) be accessible online. No date restrictions were applied.

Supplementary Table 2 Search terms combined with Boolean OR and AND operator used to identify potentially relevant literature

CATEGORY	SEARCH TERMS COMBINED WITH BOOLEAN OR OPERATOR
Discharge communication	"Discharge letter*" OR "Discharge summar*" OR "Discharge document*" OR "Discharge paper*" OR "Doctor* letter*" OR "Physician* letter*" OR "Doctor-Doctor" OR "Physician-Physician" OR "Arztbrief*" OR "Entlassbrief*" OR "Entlassungsbrief*" OR "Krankenhaus-Entlassbrief*" OR "Arzt-Arzt"
Boolean operator	"AND"
Content, structure, levels of detail preferences	"Content" OR "Structure" OR "Level of detail" OR "Abbreviation*" OR "Communication" OR "Correspondence*" OR "Satisfaction" OR "Preference*" OR "Quality" OR "Pain Point*" OR "Inhalt" OR "Struktur" OR "Detaillierungsgrad" OR "Kommunikation" OR "Korrespondenz*" OR "Zufriedenheit" OR "Präferenz*" OR "Qualität"

Supplementary Table 3 Sample of excluded articles from the literature review

REASON FOR EXCLUSION	THREE EXAMPLES OF EXCLUDED ARTICLES PER CATEGORY
Off topic	1. Epstein, D., Barak-Corren, Y., Isenberg, Y., Berger, G.: Clinical Decision Support System: A Pragmatic Tool to Improve Acute Exacerbation of COPD Discharge Recommendations. COPD: Journal of Chronic Obstructive Pulmonary Disease. (2019). https://doi.org/10.1080/15412555.2019.1593342<br>2. van Egmond, S., van Vliet, E.D., Wakkee, M., Hollestein, L.M., Pouwels, X.G.L.V., Koffijberg, H., Misirli, Y., Bakkum, R.S.L.A., Bastiaens, M.T., Kukutsch, N.A., Oosting, A.J., Plasmeijer, E.I., van Rengen, A., de Roos, K.-P., Nijsten, T.E.C., de Vries, E., de Bekker-Grob, E.W.: Efficacy, cost-minimization, and budget impact of a personalized discharge letter for basal cell carcinoma patients to reduce low-value follow-up care. PLoS One. 17, e0260978 (2022). https://doi.org/10.1371/journal.pone.0260978<br>3. Hartman, V., Campion, T.R.: A Day-to-Day Approach for Automating the Hospital Course Section of the Discharge Summary. AMIA Jt Summits Transl Sci Proc. 2022, 216–225 (2022)
Discharge general	1. Sheridan, E., Thompson, C., Pinheiro, T., Robinson, N., Davies, K., Whitmore, N.: Optimizing Transitions of Care - Hospital to Community. Healthc Q. 20, 45–49 (2017). https://doi.org/10.12927/hcq.2017.25135<br>2. Munchhof, A., Gruber, R., Lane, K.A., Bo, N., Rattray, N.A.: Beyond Discharge Summaries: Communication Preferences in Care Transitions Between Hospitalists and Primary Care Providers Using Electronic Medical Records. J Gen Intern Med. 35, 1789–1796 (2020). https://doi.org/10.1007/s11606-020-05786-2<br>3. Verhaegh, K.J., Buurman, B.M., Veenboer, G.C., de Rooij, S.E., Geerlings, S.E.: The implementation of a comprehensive discharge bundle to improve the discharge process: a quasi-experimental study. Neth J Med. 72, 318–325 (2014)
Information extraction	1. Wu, Y., Rosenbloom, S.T., Denny, J.C., Miller, R.A., Mani, S., Giuse, D.A., Xu, H.: Detecting abbreviations in discharge summaries using machine learning methods. AMIA Annu Symp Proc. 2011, 1541–1549 (2011)<br>2. Rinott, R., Torresani, M., Bertulli, R., Goldsteen, A., Casali, P., Carmeli, B., Slonim, N.: Automatic detection of inconsistencies between free text and coded data in Sarcoma discharge letters. Stud Health Technol Inform. 180, 661–666 (2012)<br>3. Kavuluru, R., Han, S., Harris, D.: Unsupervised Extraction of Diagnosis Codes from EMRs Using Knowledge-Based and Extractive Text Summarization Techniques. Adv Artif Intell. 7884, 77–88 (2013). https://doi.org/10.1007/978-3-642-38457-8_7
Communication general	1. Bell, C.M., Schnipper, J.L., Auerbach, A.D., Kaboli, P.J., Wetterneck, T.B., Gonzales, D.V., Arora, V.M., Zhang, J.X., Meltzer, D.O.: Association of communication between hospital-based physicians and primary care providers with patient outcomes. J Gen Intern Med. 24, 381–386 (2009). https://doi.org/10.1007/s11606-008-0882-8<br>2. Reponen, J., Marttila, E., Paajanen, H., Turula, A.: Extending a multimedia medical record to a regional service with electronic referral and discharge letters. J Telemed Telecare. 10 Suppl 1, 81–83 (2004). https://doi.org/10.1258/1357633042614276<br>3. Boddy, N., Barclay, S., Bashford, T., Clarkson, P.J.: How can communication to GPs at hospital discharge be improved? A systems approach. BJGP Open. 6, BJGPO.2021.0148 (2022). https://doi.org/10.3399/BJGPO.2021.0148
Patient, nurse or pharmacist	1. Siders, A.M., Peterson, M.: Increasing patient satisfaction and nursing productivity through implementation of an automated nursing discharge summary. Proc Annu Symp Comput Appl Med Care. 136–140 (1991)<br>2. NCT02673892: Patient Oriented Discharge Summary Impact Study. https://clinicaltrials.gov/show/NCT02673892. (2016)

REASON FOR EXCLUSION	THREE EXAMPLES OF EXCLUDED ARTICLES PER CATEGORY
	3. Wilcock, M., Hill, A., Wynn, A., Kelly, L.: Accuracy of pharmacist electronic discharge medicines review information transmitted to primary care at discharge. Int J Clin Pharm. 41, 820–824 (2019). https://doi.org/10.1007/s11096-019-00835-1
EHR general	1. Jayasinghe, L., Velupillai, S., Stewart, R.: Quoted text in the mental healthcare electronic record: An analysis of the distribution and content of single-word quotations. BMJ Open. 11, (2021). https://doi.org/10.1136/bmjopen-2021-049249<br>2. Häyrinen, K., Saranto, K.: The core data elements of electronic health record in Finland. Stud Health Technol Inform. 116, 131–136 (2005)<br>3. Mishra, A.K., Bhattarai, S., Bhurtel, P., Bista, N.R., Shrestha, P., Thakali, K., Banthia, P., Pathak, S.R.: Need for improvement of medical records. JNMA J Nepal Med Assoc. 48, 103–106 (2009)
Electronic vs. paper discharge summary	1. Callen, J.L., Alderton, M., McIntosh, J.: Evaluation of electronic discharge summaries: a comparison of documentation in electronic and handwritten discharge summaries. Int J Med Inform. 77, 613–620 (2008). https://doi.org/10.1016/j.ijmedinf.2007.12.002<br>2. Schabetsberger, T., Ammenwerth, E., Andreatta, S., Gratl, G., Haux, R., Lechleitner, G., Schindelwig, K., Stark, C., Vogl, R., Wilhelmy, I., Wozak, F.: From a paper-based transmission of discharge summaries to electronic communication in health care regions. Int J Med Inform. 75, 209–215 (2006). https://doi.org/10.1016/j.ijmedinf.2005.07.018<br>3. Motamedi, S.M., Posadas-Calleja, J., Straus, S., Bates, D.W., Lorenzetti, D.L., Baylis, B., Gilmour, J., Kimpton, S., Ghali, W.A.: The efficacy of computer-enabled discharge communication interventions: a systematic review. BMJ Qual Saf. 20, 403–415 (2011). https://doi.org/10.1136/bmjqs.2009.034587
Educational or feedback	1. Perel, V., Carrington, A., Janjigian, M., Schaye, V., Shur, R., Taff, J., Wagner, E., Wei, D., Yang, M., Altshuler, L.: Brief communication curriculum improves discharge summary quality. Journal of general internal medicine. 30, S51-S52 (2015)<br>2. Chua, C.E., Teo, D.B.: Writing a high-quality discharge summary through structured training and assessment. Med Educ. 57, 773–774 (2023). https://doi.org/10.1111/medu.15102<br>3. Dinescu, A., Fernandez, H., Ross, J.S., Karani, R.: Audit and feedback: an intervention to improve discharge summary completion. J Hosp Med. 6, 28–32 (2011). https://doi.org/10.1002/jhm.831
Audit	1. Gilbert, A.V., Patel, B.K., Roberts, M.S., Williams, D.B., Crofton, J.H., Morris, N.M., Wallace, J., Gilbert, A.L.: An audit of medicines information quality in electronically generated discharge summaries – Evidence to meet the australian national safety and quality health service standards. Journal of Pharmacy Practice and Research. 47, 355–364 (2017). https://doi.org/10.1002/jppr.1256<br>2. Mathur, R., Clark, R.A., Dhillon, D.P., Winter, J.H., Lipworth, B.J.: A repeat audit of hospital discharge letters in patients admitted with acute asthma. Scott Med J. 42, 19–21 (1997). https://doi.org/10.1177/003693309704200108<br>3. Eissa, A.Y.H., Mohamed Elhassan, A.Z.W., Ahmed, A.Z.H., Elgadi, A., Manhal, G.A.A., Fadul, M.H., Ahmed, M.I., Fadul, A., Mekki, I.I.: The Quality of Discharge Summaries at Al-Shaab Hospital, Sudan, in 2022: The First Cycle of a Clinical Audit. Cureus. 15, e41620 (2023). https://doi.org/10.7759/cureus.41620
No diagnosis section information	1. Spatz, H., Engel, J., Hölzel, D., Jauch, K.W.: The surgical discharge summary: a lack of substantial clinical information may affect the postop treatment of rectal cancer patients. Langenbecks Arch Surg. 386, 350–356 (2001). https://doi.org/10.1007/s004230100240

REASON FOR EXCLUSION	THREE EXAMPLES OF EXCLUDED ARTICLES PER CATEGORY
	2. Lefebvre, A., Laporte, S., Faure, S., Tiv, M., Chavanet, P., Belpois-Duchamp, C., Astruc, K., Aho-Glélé, L.S.: Information concerning multidrug-resistant bacterial colonization or infection in the medical transfer letter. Med Mal Infect. 45, 286–292 (2015). https://doi.org/10.1016/j.medmal.2015.05.008<br>3. Knaup, P., Pilz, J., Kaltschmidt, J., Ludt, S., Szecsenyi, J., Haefeli, W.E.: Standardized documentation of drug recommendations in discharge letters--a contribution to quality management in cooperative care. Methods Inf Med. 45, 336–342 (2006)

Supplementary Table 4 Reference list of included studies and their JBI quality appraisal scores

REFERENCE	JBI CRITICAL APPRAISAL SCORE
**[1]**	5 out of 8
**[2]**	6 out of 8
**[3]**	7 out of 10
**[4]**	6 out of 9
**[5]**	5 out of 6
**[6]**	10 out of 11
**[7]**	4 out of 6
**[8]**	6 out of 10
**[9]**	6 out of 8
**[10]**	5 out of 6
**[11]**	5 out of 6
**[12]**	8 out of 10
**[13]**	5 out of 8
**[14]**	4 out of 6
**[15]**	5 out of 8
**[16]**	5 out of 9
**[17]**	8 out of 10
**[18]**	7 out of 10
**[19]**	6 out of 8
**[20]**	6 out of 9
**[21]**	9 out of 11
**[22]**	5 out of 8
**[23]**	8 out of 10
**[24]**	4 out of 6
**[25]**	7 out of 11
**[26]**	6 out of 6

1. Adams, D.C., Bristol, J.B., Poskitt, K.R.: Surgical discharge summaries: improving the record. Ann R Coll Surg Engl. 75, 96–99 (1993)
2. Burrell, A., Goldszmidt, M.: Talking About Notes: Using a Design-Based Research Approach to Develop a Discharge Summary Template on a Geriatric Inpatient Unit. Can Geriatr J. 26, 326–338 (2023). https://doi.org/10.5770/cgj.26.661
3. Chatterton, B., Chen, J., Schwarz, E.B., Karlin, J.: Primary Care Physicians' Perspectives on High-Quality Discharge Summaries. J Gen Intern Med. (2023). https://doi.org/10.1007/s11606-023-08541-5
4. Dean, S.M., Gilmore-Bykovskyi, A., Buchanan, J., Ehlenfeldt, B., Kind, A.J.H.: Design and Hospitalwide Implementation of a Standardized Discharge Summary in an Electronic Health Record. Jt Comm J Qual Patient Saf. 42, 555-AP11 (2016). https://doi.org/10.1016/S1553-7250(16)30107-6
5. Glazinski, R.: Arztbriefe optimal gestalten: Leitfaden zur Erstellung qualifizierter ärztlicher Berichte in Klinik und Praxis. Books on Demand (2018)

6. Gusmeroli, M., Perks, S., Lanskey, C., Bates, N.: Australian general practitioners' views on qualities that make effective discharge communication: a scoping review. Aust J Prim Health. 29, 405–415 (2023). https://doi.org/10.1071/PY22231
7. Hammerschmidt, A.: Krankenhaus: Verfassen von Arztbriefen. Dtsch Arztebl. 119, (2022)
8. Hauschildt, K.E., Hechtman, R.K., Prescott, H.C., Iwashyna, T.J.: Hospital Discharge Summaries Are Insufficient Following ICU Stays: A Qualitative Study. Crit Care Explor. 4, e0715 (2022). https://doi.org/10.1097/CCE.0000000000000715
9. Hoffmann, M., Schwarz, C.M., Pregartner, G., Weinrauch, M., Jantscher, L., Kamolz, L., Brunner, G., Sendlhofer, G.: Attitudes of physicians towards target groups and content of the discharge summary: a cross-sectional analysis in Styria, Austria. BMJ Open. 9, e034857 (2019). https://doi.org/10.1136/bmjopen-2019-034857
10. Jatkowski, A.: Der Aufbau von Arztbriefen: Struktur und mögliche Gliederungen erklärt, https://www.arztbriefschreiben.de/aufbau-von-arztbriefen/, (2023)
11. Jatkowski, A.: So schreibt man einen optimalen Arztbrief, https://www.arztbriefschreiben.de/so-schreibt-man-einen-optimalen-arztbrief/, (2023)
12. Kergoat, M.-J., Latour, J., Julien, I., Plante, M.-A., Lebel, P., Mainville, D., Bolduc, A., Buckland, J.A.: A discharge summary adapted to the frail elderly to ensure transfer of relevant information from the hospital to community settings: a model. BMC Geriatr. 10, 69 (2010). https://doi.org/10.1186/1471-2318-10-69
13. King, M.H., Barber, S.G.: Towards better discharge summaries: brevity and structure. West Engl Med J. 106, 40–41, 55 (1991)
14. Korn, U., Beutel, S.C., Schäfer, K., van Treeck, B.: Der optimale Arztbrief - reduziert auf das Wesentliche. Hamburger Ärzteblatt. (2018)
15. Lynch, K.A., Baron, S.W., Rikin, S., Kanevsky, J., Kelly, C.B., Carrozzi, G., Wey, G., Yang, K.: Improving Resident Hospital Discharge Communication by Changing Electronic Health Record Templates to Enhance Primary Care Provider Satisfaction. Qual Manag Health Care. (2023). https://doi.org/10.1097/QMH.0000000000000417
16. Robbins, T.: Improving communication of inpatient blood transfusion events to GPs. BMJ Qual Improv Rep. 2, (2014). https://doi.org/10.1136/bmjquality.u202934.w1363
17. Schiele, F., Lemesle, G., Angoulvant, D., Krempf, M., Kownator, S., Cheggour, S., Belle, L., Ferrières, J.: Proposal for a standardized discharge letter after hospital stay for acute myocardial infarction. Eur Heart J Acute Cardiovasc Care. 9, 788–801 (2020). https://doi.org/10.1177/2048872619844444
18. Soong, C., Kurabi, B., Exconde, K., Tajammal, F., Bell, C.M.: Design of an orthopaedic-specific discharge summary. BMC Health Serv Res. 16, 545 (2016). https://doi.org/10.1186/s12913-016-1783-x
19. Sorita, A., Robelia, P.M., Kattel, S.B., McCoy, C.P., Keller, A.S., Almasri, J., Murad, M.H., Newman, J.S., Kashiwagi, D.T.: The Ideal Hospital Discharge Summary: A Survey of U.S. Physicians. J Patient Saf. 17, e637–e644 (2021). https://doi.org/10.1097/PTS.0000000000000421
20. Taylor, C.: Improving e-discharge letters for Permanent pacemaker insertions at Wansbeck General Hospital. BMJ Qual Improv Rep. 2, (2013). https://doi.org/10.1136/bmjquality.u201251.w772
21. Unnewehr, M., Schaaf, B., Marev, R., Fitch, J., Friederichs, H.: Optimizing the quality of hospital discharge summaries--a systematic review and practical tools. Postgrad Med. 127, 630–639 (2015). https://doi.org/10.1080/00325481.2015.1054256
22. van Walraven, C., Rokosh, E.: What is necessary for high-quality discharge summaries? Am J Med Qual. 14, 160–169 (1999). https://doi.org/10.1177/106286069901400403
23. Weetman, K., Spencer, R., Dale, J., Scott, E., Schnurr, S.: What makes a "successful" or "unsuccessful" discharge letter? Hospital clinician and General Practitioner assessments of the quality of discharge letters. BMC Health Serv Res. 21, 349 (2021). https://doi.org/10.1186/s12913-021-06345-z
24. Weitz, G.: Kurzanleitung zum Schreiben von Arztbriefen. In: DGIM Innere Medizin. Springer Reference Medizin, Berlin (2015)
25. Wimsett, J., Harper, A., Jones, P.: Review article: Components of a good quality discharge summary: a systematic review. Emerg Med Australas. 26, 430–438 (2014). https://doi.org/10.1111/1742-6723.12285
26. Unnewehr, M., Schaaf, B., Hendrik, F.: Arztbrief: Die Kommunikation optimieren. Dtsch Arztebl. 110, (2013)

Supplementary Table 5 Consolidated criteria for reporting qualitative research (COREQ) checklist for the focus group discussion

ITEM CATEGORY	ITEM	GUIDE QUESTIONS/DESCRIPTION	REPORTED IN SECTION
**DOMAIN 1: RESEARCH TEAM AND REFLEXIVITY**			
**Personal Characteristics**			
Interviewer/ facilitator	1	Which author/s conducted the interview or focus group?	LF and JF
Credentials	2	What were the researcher's credentials? E.g., PhD, MD	LF is a medical doctor.<br>JF holds a MPhil in Bioscience Enterprise
Occupation	3	What was their occupation at the time of the study?	LF is a doctor and researcher<br>JF is a Ph.D. candidate
Gender	4	Was the researcher male or female?	Both male
Experience and training	5	What experience or training did the researcher have?	Both researchers have extensive experience in moderating focus group discussions from their educational background and work experience as management consultants
**Relationship with participants**			
Relationship established	6	Was a relationship established prior to study commencement?	No.
Participant knowledge of the interviewer	7	What did the participants know about the researcher? e.g. personal goals, reasons for doing the research?	In advance of the FGD as well as at the beginning of the FGD, participants were informed about the educational and professional background of the researchers and were given detailed information about the research project including how prior work and how the FGD fits into the larger mixed-methods project
Interviewer characteristics	8	What characteristics were reported about the interviewer/facilitator? e.g. Bias, assumptions, reasons and interests in the research topic	Participants were informed about the personal interests of the researchers in the topic
**DOMAIN 2: STUDY DESIGN**			
**Theoretical framework**			
Methodological orientation and Theory	9	What methodological orientation was stated to underpin the study? e.g. grounded theory, discourse analysis, ethnography, phenomenology, content analysis	Structured FGD and content analysis was stated as the underlying methodological approach
**Participant selection**			

ITEM CATEGORY	ITEM	GUIDE QUESTIONS/DESCRIPTION	REPORTED IN SECTION
Sampling	10	How were participants selected? e.g., purposive, convenience, consecutive, snowball	Participants were selected via purposive sampling, based on their expertise in discharge documentation, demonstrated through teaching seminars, published articles, contributions to the digitalization of discharge communication, or over a decade of reviewing and signing off discharge summaries.
Method of approach	11	How were participants approached? e.g., face-to-face, telephone, mail, email	By email
Sample size	12	How many participants were in the study?	The FGD included seven physicians from five different hospitals and two primary care practices.
Non-participation Setting	13	How many people refused to participate or dropped out? Reasons?	One participant who agreed to attend the FGD did not show up due to unknown reasons.
Setting of data collection	14	Where was the data collected? e.g., home, clinic, workplace	Virtual focus group discussion over zoom provided by the Witten/Herdecke University
Presence of nonparticipants	15	Was anyone else present besides the participants and researchers?	No
Description of sample	16	What are the important characteristics of the sample? e.g. demographic data, date	The FGD was conducted in March 2024. The FGD included seven physicians from five different hospitals and two primary care practices. Six participants were male, one female. Every participant had at least 10 years of experience working as a physician. All participants were from different hospitals or primary care practices spread across Germany.
**Data collection**			
Interview guide	17	Were questions, prompts, and guides provided by the authors? Was it pilot tested?	Yes. A semi-structured FGD guide was used including visualizations and prompts provided on slides.
Repeat interviews	18	Were repeat interviews carried out? If yes, how many?	n/a
Audio/visual recording	19	Did the research use audio or visual recording to collect the data?	Yes. The FGD was video recorded and transcribed.
Field notes	20	Were field notes made during and/or after the interview or focus group?	Yes. Notes were written by both researchers in parallel to the recording as a redundancy.
Duration	21	What was the duration of the interviews or focus group?	See “Focus Group Discussion” section.
Data saturation	22	Was data saturation discussed?	The FGD agreed that the list of possible content elements to be tested was comprehensive and that the structure visualizations were representative and that involving additional physicians would not lead to further insights.

ITEM CATEGORY	ITEM	GUIDE QUESTIONS/DESCRIPTION	REPORTED IN SECTION
Transcripts returned	23	Were transcripts returned to participants for comment and/or correction?	We did not return transcripts to participants.
**DOMAIN 3: ANALYSIS AND FINDINGS**			
**Data analysis**			
Number of data coders	24	How many data coders coded the data?	See “Author contributions”
Description of the coding tree	25	Did the authors provide a description of the coding tree?	See “Focus Group Discussion”
Derivation of themes	26	Were themes identified in advance or derived from the data?	Derived from the data
Software	17	What software, if applicable, was used to manage the data?	MAXQDA
Participant checking	18	Did participants provide feedback on the findings?	We did not ask for feedback on the findings from participants.
**Reporting**			
Quotations presented	19	Were participant quotations presented to illustrate the themes/findings? Was each quotation identified? e.g., participant number	No, we do not present participant quotes.
Data and findings consistent	30	Was there consistency between the data presented and the findings?	Yes, data presented and findings are consistent.
Clarity of major themes	31	Were major themes clearly presented in the findings?	See “Literature Review” section under results.
Clarity of minor themes	32	Is there a description of diverse cases or a discussion of minor themes?	We did not differentiate between major and minor themes. A wide range of cases is included.

Supplementary Note 2: Translated focus group discussion guide

Disclaimer: The focus group discussion was conducted in German and the material presented in PowerPoint. This copy was translated and abstracted from PowerPoint slides.

Slide 1: Agenda

1. Introduction and Objectives
2. Structural Elements of the ideal diagnosis section
3. Content Elements of the ideal diagnosis section
4. Outlook

Slide 2: Introduction of participants and professional backgrounds in relation to discharge documentation

Slide 3-11: Background on the research project, funding, ethics approval, status quo of the research project and outlook regarding what the findings of the FGD will be used for

Slide 12: Goals for the focus group discussion

1. Verification of content and structural elements that could be part of the diagnosis section
2. Supplementation with desired "ideal" structural and content elements
3. Thus, laying the groundwork for a nationwide survey of physicians regarding their preferences

Guidelines:

1. The focus is only on the diagnosis section, not on other sections of the hospital discharge summary
2. It concerns interdisciplinary elements, not specific informational elements related to specific diagnoses
3. It is about the ideal diagnosis header, not the actual documentation practice, technical limitations of hospital information systems / practice management systems, etc.

Slide 13-24: Structural elements - For discussion:
Are there any structural elements missing?
What is the “ideal” structure?

Structural elements in the diagnosis section

Paragraphs<br>Block text<br>Tabular

Highlighting of the current treatment diagnoses<br>Highlighting of all important information<br>Highlighting the word “current” (“aktuell”)<br>Everything highlighted
Continuous numbering<br>Separate numbering of “current diagnoses” and “previous/chronic diagnoses”<br>Numbering only of the “current diagnoses”
Bullet points to detail diagnoses<br>Bullet points to list diagnoses<br>Bullet points to list and detail diagnoses
Everything listed under “diagnoses”<br>“Current diagnoses” separated from “previous/chronic diagnoses”<br>“Current” ('aktuell') under relevant diagnoses<br>“Current diagnoses”, “chronic diagnoses”, and “previous diagnoses” separated
Clinical relevance<br>Etiology/cause<br>Body/organ system
Surgeries listed with the diagnoses<br>Surgeries under a separate heading

Slide 25-34: Content elements - For discussion:
Are there any content elements missing?
Which elements should be mandatory, required, optional and which ones are not necessary?

Content elements in the diagnosis section

Name of the diagnosis
Diagnostic certainty
ICD-10 code
Severity/Stage/Classification/TNM
Localization/Extent/Pattern of involvement
Expression e.g., symptomatic, non-irritant
Course e.g., acute, chronic, recurrent

Etiology/Cause e.g., nosocomial, systemic
References to relevant findings e.g., lab tests
Histology
Brief description of the course (free text)
Date of initial diagnosis
End date of the diagnosis
Type of diagnostic finding e.g., anamnestic, incidental, invasive"
Recommendation for further procedure
Follow-up appointments
Medication
Surgery / intervention specification
Date of surgery
Operating hospital
Operation and Procedure Code
Surgery outcome
Complications
Approach e.g., conventional, minimally invasive

Substance use/toxins
Allergies/intolerances
Nursing care level classification
Pending investigations/findings
Infection/colonization with multidrug-resistant bacteria
Cardiovascular risk factors
Implants and medical devices

Slide 36

For discussion: What are potential underlying reasons for insufficient diagnosis sections?
e.g., lack of training, time pressure, insufficient review by chief/senior physicians…

Slide 37

Thank you and closing

Supplementary Table 6 Checklist for reporting results of internet e-surveys (CHERRIES)

ITEM CATEGORY	ITEM	EXPLANATION	REPORTED IN SECTION
**DESIGN**			
Describe survey design	1	Describe target population, sample frame. Is the sample a convenience sample? (In "open" surveys this is most likely.)	see section "2.4 Online survey"
**IRB (Institutional Review Board) APPROVAL AND INFORMED CONSENT PROCESS**			
IRB approval	2	Mention whether the study has been approved by an IRB.	see section "2.1 Study design"
Informed consent	3	Describe the informed consent process. Where were the participants told the length of time of the survey, which data were stored and where and for how long, who the investigator was, and the purpose of the study?	see section "2.4 Online survey"
Data protection	4	If any personal information was collected or stored, describe what mechanisms were used to protect unauthorized access.	We did not collect any personal information that could be tied back to individual respondents. Data was stored solely on the Witten/Herdecke University server.
**DEVELOPMENT AND PRE-TESTING**			
Development and testing	5	State how the survey was developed, including whether the usability and technical functionality of the electronic questionnaire had been tested before fielding the questionnaire.	After drafting, it was pretested with four physicians and four researchers. Feedback from the pretest led to revisions in question wording and the removal of two questions.
**RECRUITMENT PROCESS AND DESCRIPTION OF THE SAMPLE HAVING ACCESS TO THE QUESTIONNAIRE**			
Open survey versus closed survey	6	An "open survey" is a survey open for each visitor of a site, while a closed survey is only open to a sample which the investigator knows (password-protected survey).	see section "2.4 Online survey"
Contact mode	7	Indicate whether or not the initial contact with the potential participants was made on the Internet. (Investigators may also send out questionnaires by mail and allow for Web-based data entry.)	see section "2.4 Online survey"

Advertising the survey	8	How/where was the survey announced or advertised? Some examples are offline media (newspapers), or online (mailing lists – If yes, which ones?) or banner ads (Where were these banner ads posted and what did they look like?). It is important to know the wording of the announcement as it will heavily influence who chooses to participate. Ideally the survey announcement should be published as an appendix.	see section “2.4 Online survey”<br><br>To obtain a broad sample of German physicians, we used a modified Dillmann technique to recruit physicians via two main channels. First, outpatient physicians were recruited via E-Mail through cooperating regional chapters of the General Practitioners’ Associations (“Hausärztinnen- und Hausärzteverband”) and cooperating Associations of Statutory Health Insurance Physicians (“Kassenärztliche Vereinigungen”). Second, participants were contacted via the physician mailing list of Helios Healthcare, the largest hospital group in Germany comprising 87 hospitals of different sizes and specializations.<br><br>The announcement used in the emails read as follows (translated English version): “Dear [PHYSICIAN’S NAME], As part of a research project at the Faculty of Health at Witten/Herdecke University, we are investigating physicians' preferences regarding the structure and content of the diagnosis section in hospital discharge summaries. Our goal is to develop a standardized digital diagnosis section, similar to the national medication plan (“Medikationsplan”), that is tailored to the needs of physicians and everyday clinical practice. We would greatly appreciate it if you could share your personal preferences for the diagnosis section by participating in our 10-minute scientific survey: https://limesurvey.uni-wh.de/index.php/956621. Thank you very much for your support. With kind regards, Your research team.” Full names and pictures of the research team were provided in the email footer.
**SURVEY ADMINISTRATION**			

Web/E-Mail	9	State the type of e-survey (e.g., one posted on a Web site, or one sent out through e-mail). If it is an e-mail survey, were the responses entered manually into a database, or was there an automatic method for capturing responses?	The survey was administered solely through the web. We utilized the online survey tool LimeSurvey (vers. 6.4) provided by the Witten/Herdecke University to create and launch the survey.
Context	10	Describe the Web site (for mailing list/newsgroup) in which the survey was posted. What is the Web site about, who is visiting it, what are visitors normally looking for? Discuss to what degree the content of the Web site could pre-select the sample or influence the results. For example, a survey about vaccination on an anti immunization Web site will have different results from a Web survey conducted on a government Web site	n/a
Mandatory/voluntary	11	Was it a mandatory survey to be filled in by every visitor who wanted to enter the Web site, or was it a voluntary survey?	see section “2.4 Online survey”
Incentives	12	Were any incentives offered (e.g., monetary, prizes, or non-monetary incentives such as an offer to provide the survey results)?	No incentives were offered.
Time/date	13	In what timeframe were the data collected?	see section “2.4 Online survey”. The data was collected between April and July 2024.
Randomization of items or questionnaires	14	To prevent biases items can be randomized or alternated.	Answer choices were randomized for all questions, except where doing so would impact readability (e.g., in questions such as how many years you have been working as a physician). The Supplement includes the translated version of the survey questionnaire, indicating which questions had randomized answer choices.
Adaptive questioning	15	Use adaptive questioning (certain items, or only conditionally displayed based on responses to other items) to reduce number and complexity of the questions.	Adaptive questioning was used for the questions that only pertained to hospital physicians: question 5 on audiences of the diagnosis section and question 11 on underlying reasons for insufficient diagnosis section as well as demographic questions 28-30.

Number of items	16	What was the number of questionnaire items per page? The number of items is an important factor for the completion rate.	The final version of the survey included 31 questions divided into five sections: (1) usage of the diagnosis section (2), satisfaction with and importance of the diagnosis section, (3) content preferences, (4) structural preferences, and (5) personal and professional background. See the translated version of the survey questionnaire for the split by pages. Across all questions the survey consisted of 120 items.
Number of screens (pages)	17	Over how many pages was the questionnaire distributed? The number of items is an important factor for the completion rate.	The questionnaire was split onto different pages by section as outline above. Including the introduction page, the questionnaire was distributed over 6 pages.
Completeness check	18	It is technically possible to do consistency or completeness checks before the questionnaire is submitted. Was this done, and if "yes", how (usually JAVAScript)? An alternative is to check for completeness after the questionnaire has been submitted (and highlight mandatory items). If this has been done, it should be reported. All items should provide a non-response option such as "not applicable" or "rather not say", and selection of one response option should be enforced.	We utilized soft- forced-response (i.e., reminders to provide complete answers) for most questions, therefore a non-response option was included for most questions. Only questions 13 and 21 were forced response questions. In cases where totals differ due to respondents not answering a particular (sub-)question, the number of respondents per (sub-) question is described.
Review step	19	State whether respondents were able to review and change their answers (e.g., through a Back button or a Review step which displays a summary of the responses and asks the respondents if they are correct).	Respondents were able to review and change their answers through a Back button.
**RESPONSE RATES**			

Unique site visitors	20	If you provide view rates or participation rates, you need to define how you determined a unique visitor. There are different techniques available, based on IP addresses or cookies or both.	see section “2.4 Online survey” and Figure 2.
View rate (Ratio of unique survey visitors/unique site visitors)	21	Requires counting unique visitors to the first page of the survey, divided by the number of unique site visitors (not page views!). It is not unusual to have view rates of less than 0.1 % if the survey is voluntary.	n/a
Participation rate (ratio of those who agreed to participate/unique first survey page visitors)	22	Count the unique number of people who filled in the first survey page (or agreed to participate, for example by checking a checkbox), divided by visitors who visit the first page of the survey (or the informed consents page, if present). This can also be called “recruitment” rate.	see section “2.4 Online survey” and Figure 2.
Completion rate (ratio of users who finished the survey/users who agreed to participate)	23	The number of people submitting the last questionnaire page, divided by the number of people who agreed to participate (or submitted the first survey page). This is only relevant if there is a separate “informed consent” page or if the survey goes over several pages. This is a measure for attrition. Note that “completion” can involve leaving questionnaire items blank. This is not a measure for how completely questionnaires were filled in. (If you need a measure for this, use the word “completeness rate”.)	see section “2.4 Online survey” and Figure 2.
**PREVETING MULTIPLE ENTRIES FROM THE SAME INDIVIDUAL**			

Cookies used	24	Indicate whether cookies were used to assign a unique user identifier to each client computer. If so, mention the page on which the cookie was set and read, and how long the cookie was valid. Were duplicate entries avoided by preventing users access to the survey twice; or were duplicate database entries having the same user ID eliminated before analysis? In the latter case, which entries were kept for analysis (e.g., the first entry or the most recent)?	We chose not to use cookies, as this could unintentionally exclude physicians sharing a computer (oftentimes the case in hospital and practice settings) from participating in the survey.
IP check	25	Indicate whether the IP address of the client computer was used to identify potential duplicate entries from the same user. If so, mention the period of time for which no two entries from the same IP address were allowed (e.g., 24 hours). Were duplicate entries avoided by preventing users with the same IP address access to the survey twice; or were duplicate database entries having the same IP address within a given period of time eliminated before analysis? If the latter, which entries were kept for analysis (e.g., the first entry or the most recent)?	see section "2.4 Online survey" and Figure 2 for the data cleaning approach following best practices.<br><br>We checked for any cases with identical demographic data. No such cases were found.
Log file analysis	26	Indicate whether other techniques to analyze the log file for identification of multiple entries were used. If so, please describe.	n/a
Registration	27	In "closed" (non-open) surveys, users need to login first and it is easier to prevent duplicate entries from the same user. Describe how this was done. For example, was the survey never displayed a second time once the user had filled it in, or was the username stored together with the survey results and later eliminated? If the latter, which entries were kept for analysis (e.g., the first entry or the most recent)?	n/a
**ANALYSIS**			

Handling of incomplete responses	28	Were only completed questionnaires analyzed? Were questionnaires which terminated early (where, for example, users did not go through all questionnaire pages) also analyzed?	see ‘Online survey’ and ‘Figure 7’
Questionnaires submitted with atypical timestamp	29	Some investigators may measure the time people needed to fill in a questionnaire and exclude questionnaires that were submitted too soon. Specify the timeframe that was used as a cut-off point and describe how this point was determined.	see ‘Online survey’ and ‘Figure 7’. Of all participants that completed the survey, those with a relative completion time below the threshold of the fastest 2% (<7m38s) were excluded (n = 12)
Statistical correction	30	Indicate whether any methods such as weighting of items or propensity scores have been used to adjust for the non-representative sample; if so, please describe the methods.	n/a

Supplementary Note 3 Translated survey questionnaire

Disclaimer: The questionnaire was presented to respondents in German. This copy was translated.

The Diagnosis Section in Hospital Discharge Summaries - Expectations and Preferences of German Physicians

Introduction ---

Thank you very much for your interest in this study!

The hospital discharge summary (“KH-Entlassbrief”) is a central communication tool between hospitals and the outpatient sector. However, there is currently a lack of standardization that allows for the automatic transfer of important information into the IT systems of subsequent care providers.

In this study, we aim to determine the preferences of physicians in Germany regarding the overall structure, content, and levels of detail of the diagnosis section in discharge summaries.

The goal of the research is to define the content requirements for a standardized digital diagnosis section, similar to the nationwide medication plan (“Medikationsplan”). This will be incorporated into the programmatic architecture of the KV-MIO "KH-Entlassbrief" and thus be interoperable with the electronic patient record.

This digital diagnosis section will be based on the preferences of the medical profession and aligned with clinical reality and the everyday care environment in practice.

Your responses will help us take into account the needs of both the authors in the hospital and the recipients in the outpatient sector.

The survey will take approximately 10 minutes to complete.

If you have any questions, feel free to contact us via email [Contact information]

Thank you!

Ethics Committee Approval ---

This study was reviewed by the Ethics Committee of the University of Witten/Herdecke e.V., and no objections were raised (Approval No. S-311/2023). Below, you will find all relevant information regarding data processing in the context of this study as well as your rights as a participant. Your electronic consent is required to participate in the study.

Consent to Data Collection in the Context of the Study ---

Responsible Entity / Conducting Department	Study Coordination	Conducting Person
[Name and contact details]	[Name and contact details]	[Name and contact details]

I have been informed that data from the participants will be processed anonymously as part of this online survey. I have been told the purpose, extent, legal basis, and duration of data storage. I have received a corresponding information sheet.

I understand that consent is voluntary. I am aware that a request for deletion cannot be fulfilled, as the data cannot be uniquely assigned to me as an individual.

View privacy policy here (LINK).

This survey contains 31 questions.

Introduction --

⚠ IMPORTANT CLARIFICATION: What do we mean by "Diagnosis Section"?

The "Diagnosis Section" refers to the part at the beginning of the hospital discharge summary listed under "Diagnoses." In current clinical practice, this section includes not only diagnosis names and ICD-10 codes but also important information about the course of the diagnoses.

1. I work as:

   ❑ a hospital physician (inpatient) – all specialties

   ❑ an outpatient physician (ambulatory)

2. Structural and content standards in the diagnosis section would facilitate the accurate and unambiguous transmission of treatment-related information

Strongly disagree	Disagree	Neutral	Agree	Strongly agree
❑	❑	❑	❑	❑

Satisfaction with and Importance of Diagnosis Sections ---

⚠ Please rate based on the average hospital discharge summaries you see daily.

3. How satisfied are you with the following sections in hospital discharge summaries?
   Please select the appropriate answer for each point:

	Strongly dissatisfied	Dissatisfied	Neutral	Satisfied	Very satisfied
Overall hospital discharge summary (structure and content)	❑	❑	❑	❑	❑
Diagnosis section<br>History	❑	❑	❑	❑	❑
Medication plan (discharge medication)	❑	❑	❑	❑	❑
Findings / Results	❑	❑	❑	❑	❑
Procedures / Recommendations for further treatment	❑	❑	❑	❑	❑
Summary (epicrisis)	❑	❑	❑	❑	❑

4. How important is the diagnosis section in the hospital discharge summary for you for the subsequent care of the patient?

Very unimportant	Unimportant	Neutral	Important	Very important
❑	❑	❑	❑	❑

5. How important is the diagnosis section in the hospital discharge summary for you to record information for the following recipients?
Answer this question only if the following condition is met: The answer was 'as a hospital physician (inpatient) – all specialties' in question [Q00001] (I work)
Answer choice order randomized

	Very unimportant	Unimportant	Neutral	Important	Very important
Myself	❑	❑	❑	❑	❑
Subsequent care providers	❑	❑	❑	❑	❑
Medical service of health insurance (“MDK”)	❑	❑	❑	❑	❑
Colleagues in the hospital	❑	❑	❑	❑	❑
Patients	❑	❑	❑	❑	❑
Medical coders in the hospital (billing)	❑	❑	❑	❑	❑

6. How important are the following aspects of diagnosis sections to you in general?
Please select the appropriate answer for each point:

	Very unimportant	Unimportant	Neutral	Important	Very important
Content overall	❑	❑	❑	❑	❑
Completeness	❑	❑	❑	❑	❑
Relevance/timeliness	❑	❑	❑	❑	❑
Correctness	❑	❑	❑	❑	❑
Understandability for physicians	❑	❑	❑	❑	❑
Understandability for patients	❑	❑	❑	❑	❑
High level of detail	❑	❑	❑	❑	❑
Structure overall	❑	❑	❑	❑	❑
Layout	❑	❑	❑	❑	❑
Typography e.g., bolding, bullet points	❑	❑	❑	❑	❑
Content structuring e.g., headings, sorting	❑	❑	❑	❑	❑
Interoperability with my software	❑	❑	❑	❑	❑

7. How satisfied are you in general with the following aspects of diagnosis sections?
Please select the appropriate answer for each point:

	Very dissatisfied	Dissatisfied	Neutral	Satisfied	Very satisfied
Content overall	❑	❑	❑	❑	❑
Completeness	❑	❑	❑	❑	❑
Relevance/timeliness	❑	❑	❑	❑	❑
Correctness	❑	❑	❑	❑	❑
Understandability for physicians	❑	❑	❑	❑	❑
Understandability for patients	❑	❑	❑	❑	❑
High level of detail	❑	❑	❑	❑	❑
Structure overall	❑	❑	❑	❑	❑
Layout	❑	❑	❑	❑	❑
Typography e.g., bolding, bullet points	❑	❑	❑	❑	❑
Content structuring e.g., headings, sorting	❑	❑	❑	❑	❑
Interoperability with my software	❑	❑	❑	❑	❑

8. What percentage of diagnosis sections you receive are you completely satisfied with?
   0% = not satisfied with any diagnosis sections.
   100% = completely satisfied with all diagnosis sections.
   Only whole numbers can be entered in these fields in 10 percentage point increments.
   Please enter your answer(s) here:

From basic care hospitals (<250 beds)	0% -------------------●----------------------- 100%
From tertiary care hospitals (e.g., teaching hospitals)	0% -------------------●----------------------- 100%

9. In general, the level of detail in the diagnosis section is:
   Please select the appropriate answer for each point:

Too low	Low	Appropriate	High	Too high
❑	❑	❑	❑	❑

10. Please indicate how much you agree with each of the following statements about abbreviations in diagnosis sections.
    Please select the appropriate answer for each point:

	Strongly disagree	Disagree	Neutral	Agree	Strongly agree
No abbreviations should be used in diagnosis sections, not even commonly known ones like "Z.n.," "BMI," or "bds."	❑	❑	❑	❑	❑
No field-specific abbreviations should be used in diagnosis sections, such as "LAHB," "TIA," or "EGD."	❑	❑	❑	❑	❑
The use of abbreviations improves the efficiency of writing and reading diagnosis sections.	❑	❑	❑	❑	❑
The risk of miscommunication due to abbreviations is outweighed by their time- and space-saving benefits in diagnosis sections.	❑	❑	❑	❑	❑

11. How relevant do you consider the following potential causes of inadequate diagnosis sections in hospital discharge summaries?
    Answer this question only if the following condition is met: (Q00001.NAOK == "AO01")
    Answer choices randomized

	Not at all relevant	Somewhat relevant	Neutral	Relevant	Very relevant
Deficient admission documentation	❑	❑	❑	❑	❑
Poor German language skills of the authors	❑	❑	❑	❑	❑
Lack of training	❑	❑	❑	❑	❑
Not authored by the treating physician	❑	❑	❑	❑	❑
Lack of pre-printed text modules	❑	❑	❑	❑	❑
Deficiencies in hospital information system / documentation software	❑	❑	❑	❑	❑
Copy-pasting from prior discharge summaries without review	❑	❑	❑	❑	❑
Time pressure	❑	❑	❑	❑	❑
Lack of general guidelines/conventions	❑	❑	❑	❑	❑
Insufficient review by chief/senior physicians	❑	❑	❑	❑	❑
Lack of training	❑	❑	❑	❑	❑
Incomplete records	❑	❑	❑	❑	❑
Special requirements/preferences of superiors	❑	❑	❑	❑	❑

Content preferences ---

12. What do you use the diagnosis section for?
    Select all applicable options:
    Answer choices randomized

    - ❑ Transfer diagnoses into IT system
    - ❑ Gain a brief overview of the current course of illness
    - ❑ Gain a comprehensive picture of the patient
    - ❑ Gain a detailed overview of the current course of illness
    - ❑ Other: *please specify* ______________________

13. What content elements should each current treatment diagnosis (primary diagnosis) in the diagnosis section include?

	Mandatory (must)	Required, if available (should)	Desirable, if available (can)	Not necessary
Diagnosis information				
Name of the diagnosis	❑	❑	❑	❑
Diagnostic certainty	❑	❑	❑	❑
ICD-10 code	❑	❑	❑	❑
Diagnosis specification				
Severity/Stage/Classification/TNM	❑	❑	❑	❑
Localization/Extent/Pattern of involvement	❑	❑	❑	❑
Expression e.g., symptomatic, non-irritant	❑	❑	❑	❑
Course e.g., acute, chronic, recurrent	❑	❑	❑	❑
Etiology/Cause e.g., nosocomial, systemic	❑	❑	❑	❑
References to relevant findings e.g., lab tests	❑	❑	❑	❑
Histology	❑	❑	❑	❑
Brief description of the course (free text)	❑	❑	❑	❑
Diagnosis history and diagnostic process				
Date of initial diagnosis	❑	❑	❑	❑
End date of the diagnosis	❑	❑	❑	❑
Type of diagnostic finding e.g., anamnestic, incidental, invasive"	❑	❑	❑	❑
Diagnosis recommendation				
Recommendation for further procedure	❑	❑	❑	❑
Follow-up appointments / Tumor board date	❑	❑	❑	❑
Medication (changes/critical)	❑	❑	❑	❑
Therapy	❑	❑	❑	❑
Surgery / intervention specification	❑	❑	❑	❑
References to relevant surgeries/interventions				
Date of surgery	❑	❑	❑	❑
Operating hospital	❑	❑	❑	❑
Operation and Procedure Code	❑	❑	❑	❑
Surgery outcome e.g., incomplete excision	❑	❑	❑	❑
Complications	❑	❑	❑	❑
Approach e.g., conventional, minimally invasive	❑	❑	❑	❑

14. What information, beyond the current treatment diagnoses (primary diagnoses), should be included in the diagnosis section?
Answer choices randomized

	Mandatory (must)	Required, if available (should)	Desirable, if available (can)	Not necessary
Substance use/toxins	❑	❑	❑	❑
Allergies/intolerances	❑	❑	❑	❑
Family medical history	❑	❑	❑	❑
Nutritional status (weight, BMI, LDL-C)	❑	❑	❑	❑
DNI/DNR/organ donation status	❑	❑	❑	❑
Nursing care level classification	❑	❑	❑	❑
Pending investigations/findings	❑	❑	❑	❑
Infection/colonization with multidrug-resistant bacteria	❑	❑	❑	❑
Cardiovascular risk factors	❑	❑	❑	❑
Implants and medical devices	❑	❑	❑	❑

Content preferences ---

15. Which layout do you prefer for the diagnosis section?
Please select one of the following options:
Answer choices randomized

❑	Block text
❑	Tabular
❑	Paragraphs

16. What numbering of diagnoses do you prefer?
Please select one of the following options:
Answer choices randomized

❑	Continuous numbering
❑	No numbering
❑	Numbering only of the current treatment diagnoses
❑	Separate numbering of current treatment diagnoses and chronic/prevoius diagnoses

17. How should diagnoses be listed/sorted?
Please select one of the following options:
Answer choices randomized
    - ❑ Clinical relevance
    - ❑ Body / Organ system
    - ❑ Etiology / Cause
    - ❑ Billing relevance
    - ❑ Alphabetical order

18. What way of structuring diagnoses do you prefer?
Please select one of the following options:
Answer choices randomized

❑	“Currently” ("Aktuell") under relevant diagnoses
❑	"Current treatment diagnoses", "Chronic diagnoses", and "Past medical history" separated
❑	Current treatment diagnoses" separated from "Past medical history and chronic diagnoses"
❑	Everything listed under "Diagnoses"

19. What format do you prefer for structuring procedures/surgeries?
Please select one of the following options:
Answer choices randomized

❑	Performed procedures / surgeries under a separate heading
❑	Performed procedures in parentheses after the diagnosis
❑	Performed procedures / surgeries listed with the diagnoses

20. Which representation do you prefer to highlight the relevant aspects of the current hospital stay?
Please select one of the following options:
Answer choices randomized

❑	Highlighting the current treatment diagnoses
❑	Highlighting the word “currently” ("aktuell")
❑	"Checkbox" indicating diagnoses treated in current stay
❑	Highlighting of all important information

21. What interactive elements would you like for reading a digital diagnosis section?
Please select all of the options that apply:
Answer choices randomized

❑ Expandable elements
❑ Tooltip
❑ QR codes linking to additional information
❑ Links
❑ Search function
❑ Filter function
❑ Color highlighting
❑ Automatic translation into various languages
❑ Other, please specify:____________________
❑ No interactive elements *(disables all the above)*

Personal and professional background ---

22. With which gender do you identify?
Please select one of the options:

❑ Male
❑ Female
❑ Diverse

23. Which age group are you in?
Please select one of the options:
- ❑ Under 26 years old
- ❑ 26 – 35 years old
- ❑ 36 – 45 years old
- ❑ 46 – 55 years old
- ❑ 56 – 65 years old
- ❑ 66 years old or older

24. Please specify your (primary) specialty ("Facharzt")?
Please select one of the options:
- ❑ Anatomy
- ❑ Anesthesiology
- ❑ Biochemistry
- ❑ Cardiac Surgery
- ❑ Child and Adolescent Psychiatry and Psychotherapy
- ❑ Dermatology and Venereology
- ❑ Forensic Medicine
- ❑ General Medicine
- ❑ General Surgery
- ❑ Gynecology and Obstetrics
- ❑ Human Genetics
- ❑ Hygiene and Environmental Medicine
- ❑ Internal and General Medicine
- ❑ Internal Medicine
- ❑ Internal Medicine and Angiology
- ❑ Internal Medicine and Cardiology
- ❑ Internal Medicine and Endocrinology and Diabetology
- ❑ Internal Medicine and Gastroenterology
- ❑ Internal Medicine and Hematology and Oncology
- ❑ Internal Medicine and Nephrology
- ❑ Internal Medicine and Pulmonology

- ❑ Internal Medicine and Rheumatology
- ❑ Laboratory Medicine
- ❑ Microbiology, Virology, and Infection Epidemiology
- ❑ Neurosurgery
- ❑ Neurology
- ❑ Neuropathology
- ❑ Nuclear Medicine
- ❑ Occupational Medicine
- ❑ Ophthalmology
- ❑ Oral and Maxillofacial Surgery
- ❑ Orthopedics and Trauma Surgery
- ❑ Otorhinolaryngology (ENT – Ear, Nose, and Throat)
- ❑ Pathology
- ❑ Pediatric Surgery
- ❑ Pediatrics
- ❑ Pharmacology and Toxicology
- ❑ Physical and Rehabilitative Medicine
- ❑ Physiology
- ❑ Plastic and Aesthetic Surgery
- ❑ Psychiatry and Psychotherapy
- ❑ Psychosomatic Medicine and Psychotherapy
- ❑ Public Health
- ❑ Radiation Therapy
- ❑ Radiology
- ❑ Speech, Voice, and Pediatric Hearing Disorders
- ❑ Thoracic Surgery
- ❑ Transfusion Medicine
- ❑ Vascular Surgery
- ❑ Visceral Surgery

25. How many years have you been practicing as a physician?
Please select one of the options:
    - ❑ Less than 5 years

- ❑ 6– 10 years
- ❑ 11 – 20 years
- ❑ 21 – 30 years
- ❑ 31 years or more

26. In which federal state do you practice?
    Please select one of the options:
    - ❑ Baden-Württemberg
    - ❑ Bavaria
    - ❑ Berlin
    - ❑ Brandenburg
    - ❑ Bremen
    - ❑ Hamburg
    - ❑ Hesse
    - ❑ Mecklenburg-Vorpommern
    - ❑ Lower Saxony
    - ❑ North Rhine-Westphalia
    - ❑ Rhineland-Palatinate
    - ❑ Saarland
    - ❑ Saxony
    - ❑ Saxony-Anhalt
    - ❑ Schleswig-Holstein
    - ❑ Thuringia

27. Where do you work as a physician? I work in a city / municipality with:
    Please select one of the options:
    - ❑ Under 5,000 inhabitants
    - ❑ 5,000 – 20,000 inhabitants
    - ❑ 20,001 – 100,000 inhabitants
    - ❑ 100,001 – 500,000 inhabitants
    - ❑ More than 500,000 inhabitants

28. What type of hospital are you working in (size)?
Answer this question only if the following condition is met: ((Q00001.NAOK == "AO01"))
Please select only one of the following answers:
- ❑ Basic care (<250 beds)
- ❑ Secondary care (251 – 800 beds)
- ❑ Tertiary (>800 beds)

29. Are you working in a teaching hospital?
Answer this question only if the following condition is met: ((Q00001.NAOK == "AO01"))
Please select only one of the following answers:
- ❑ Yes
- ❑ No

30. What type of hospital are you working in (ownership)?
Answer this question only if the following condition is met: ((Q00001.NAOK == "AO01"))
Please select only one of the following answers:
- ❑ Public
- ❑ Non-profit
- ❑ Privat
- ❑ Other, please specify: _____________

31. Would you like to share any additional thoughts on diagnosis sections in hospital discharge summaries or on the content of this study?
Please enter your response here (optional). Free text (unlimited).

____

Thank you for your participation! Your responses have been successfully recorded.

Supplementary Table 7 Extracted data from articles included in the literature scoping review

TITLE	YEAR	COUNTRY	MAIN OBJECTIVE	SPECIALTY/ DISEASE	METHOD (SAMPLE SIZE)	DIAGNOSIS SECTION SPECIFIC RESULTS	TEMPLATE?
Surgical discharge summaries: improving the record.	1993	United Kingdom	To gain insight on views of GPs on relative importance of various aspects of surgical discharge summaries	Surgical	Survey (118 GPs)	- Put into structured format<br>- Diagnosis most important element	No
Talking About Notes: Using a Design-Based Research Approach to Develop a Discharge Summary Template on a Geriatric Inpatient Unit.	2023	Canada	To assess what content should be included in a geriatric discharge summary	Geriatric	Mixed-methods (iterative consensus based template development)	- Start with most important active issue (usually same as “most responsible diagnosis”)<br>- Use a separate paragraph for each diagnosis	Yes
Primary Care Physicians' Perspectives on High-Quality Discharge Summaries.	2023	United States	To explore GPs perspectives on characteristics of high-quality discharge summaries	Generic	Semi-structured individual interviews (20 GPs)	- Create a header to list incidental findings<br>- Include a brief to-do list associated with diagnoses<br>- Exclude irrelevant details (e.g., plans not relevant to outpatient care)	No
Design and Hospitalwide Implementation of a Standardized Discharge Summary in an Electronic Health Record.	2016	United States	To design and implement hospital wide standardized discharge summary	Generic	Quasi-experimental: Implementation of new standard discharge template developed by task force	- Include a primary and secondary discharge diagnosis, and discharge disposition<br>-Include operative and other procedures performed<br>- Include active issues requiring follow-up (issue, what is needed, associated appointments)<br>- Standardized template increased satisfaction of authors and receiving physicians	Yes

Arztbriefe optimal gestalten: Leitfaden zur Erstellung qualifizierter ärztlicher Berichte in Klinik und Praxis		German y	To provide general guidelines on structure and content of discharge summaries	Generic	Expert opinion	- Start with the main diagnosis, followed by secondary diagnoses and procedures including dates<br>'- Code the diagnoses using ICD-10 and the procedures using the current OPS codes	No
Australian general practitioners' views on qualities that make effective discharge communication: a scoping review.	2023	Australi a	To map and discuss Australian general practitioners' views on the qualities that make up effective discharge communication	Generic	Systematic (scoping) review	- GPs prefer diagnosis specific information<br>- Use diagnosis specific templates to promt clinicians to add pertinent information	No
Krankenhaus: Verfassen von Arztbriefen		German y	To provide practical advice on what information to include in discharge summaries	Generic	Expert opinion	- Group related diagnoses together where appropriate. Previous diagnoses from other medical reports should be critically reviewed, not blindly copied.<br>- Include interventions with the corresponding dates	No
Hospital Discharge Summaries Are Insufficient Following ICU Stays: A Qualitative Study.	2022	United States	To understand what additional information GPs desire in discharge summaries to support patients' recovery following critical illness	Intensive care	Modified Rigorous and Accelerated Data Reduction (RADaR) technique	- Include active and resolved hospital problems<br>- List reason for ICU admission, maximum life support required, and any complications	No
Attitudes of physicians towards target groups and content of the discharge summary: a cross-sectional analysis in Styria, Austria.	2019	Austria	To investigate discharge summary target audience and which contents are necessary to ensure a safe treatment	Generic	Cross-sectional online survey (1,060 physicians)	- 100% agreement that diagnosis is mandatory<br>- Lack of uniform structure is an issue	No
Der Aufbau von Arztbriefen: Struktur und mögliche Gliederungen erklärt	2024	German y	To provide practical guidance on structure of discharge summaries	Generic	Expert opinion	- List current diagnoses first, then additional/previous diagnoses<br>- Add ICD-10 codes<br>- After diagnoses, include surgical, oncological, or similar treatment details.	No

So schreibt man einen optimalenArztbrief		German y	To provide advice and guidance on how to write a discharge summary	Generic	Expert opinion	- Remove old or irrelevant entries, eliminate duplicates, and group related diagnoses together.<br>- Place the most important diagnoses at the top and move older ones under "transferred diagnoses."<br>- Specify conditions and use classifications or scores for accuracy.<br>- Use numbering and indentation for structuring<br>- List other diagnoses under a separate heading	No
A discharge summary adapted to the frail elderly to ensure transfer of relevant information from the hospital to community settings: a model.	2020	Canada	To develop a discharge summary adapted to frail elderly patient	Geriatric	Modified Delphi-Method (21 physicians, 10 pharmacists)	- Separate into active and non-active diagnoses.<br>- Include: admission diagnosis, main diagnosis (es), other diagnoses and problem(s), complications, treatments, special investigations.<br>- Specify if: allergy, chronic pain, tobacco, alcohol.<br>- Provide details on diagnoses	Yes
Towards better discharge summaries: brevity and structure.	1991	United Kingdo m	To assess GP's askribed importance to format and content of discharge summaries.	Generic	Survey (99 GPs)	- Add date of initial diagnosis<br>- List diagnoses in form of prioritized problem list<br>- Ensure brevity and structure (number diagnoses)	No
Der optimale Arztbrief – reduziert auf das Wesentliche	2018	German y	To outline what should be included in discharge summaries based on GP needs	Generic	Expert opinion	- Use an interdisciplinary uniform, standardized structure<br>- Include ICD-10 codes	No
Improving Resident Hospital Discharge Communication by Changing Electronic Health Record Templates to Enhance Primary Care Provider Satisfaction.	2023	United States	To develop and enhance electronic discharge summary template to improve GP satisfaction with written discharge summaries	Generic	Survey (30 physicians)	- Do not present the chief complaints as diagnoses<br>- Indicate diagnostic tests conducted during hospitalization<br>- List diagnoses, not symptoms<br>- Significant increase in GP satisfaction after introduction of template	Yes

Improving communication of inpatient blood transfusion events to GPs.	2014	United Kingdo m	To design a section in discharge summaries for the documentation of blood transfusion events	Blood transfusion	Quasi-experimental: Implementatio n of new template for documenting blood transfusion	- Include information on blood transfusion: date of transfusion, whether or not a transfusion reaction occured, how many transfusions were given, why the transfusion occured<br>- 30 percentage point increase in discharge summaries that included blood transfusion events after introduction of template	Yes
Proposal for a standardized discharge letter after hospital stay for acute myocardial infarction	2020	France	To create a consensus standard discharge letter template for AMI	Acute Myocardial Infarction (AMI)	3-step Delphi method (32 physicians)	- Include CVRF, cardiovascular history, reasons for admission, main diagnosis, additional diagnosis;<br>- Include admission and target HbA1c	Yes
Design of an orthopaedic-specific discharge summary.	2016	United States	To design an orthopedic-specific discharge summary	Orthopedics	Semi-structured interviews (17 patients and physicians)	- Include admitting and final diagnosis	Yes
The Ideal Hospital Discharge Summary: A Survey of U.S. Physicians.	2021	United States	To assess physicians' perspectives about discharge summaries and the differences between inpatient and outpatient physicans	Generic	Survey (1,600 physicians)	- List of diagnoses and problems most important<br>- Consider patients as important stakeholders	No
Improving e-discharge letters for Permanent pacemaker insertions at Wansbeck General Hospital.	2013	United Kingdo m	To establish the information required in all discharge summaries for pacemaker implantation and testing effect of guideline implementatoin	Permanent pacemaker insertion	Mixed-methods. Expert panel to write guideline and quasi-experimental testing prior/post implementatio n	- Include date and indication for insertion, pacemaker type, make and model, access route, complications, chest x-ray and device check results, and follow-up details<br>- Dramatic improvement in discharge letters that included necessary information after introduction of the template	Yes

Optimizing the quality of hospital discharge summaries--a systematic review and practical tools.	2015	German y	To identify possibilities for improving the quality of discharge summaries	Generic	Systematic (scoping) review	- Ensure all of diagnoses are complete, up-to-date, and accurate (including stages and classifications)<br>- Ensure the list of diagnoses is structured and clear<br>- Ensure diagnosis section is free from irrelevant content	No
What is necessary for high-quality discharge summaries?	1999	United States	To determine what physicians percieve to be necessary for high-quality discharge summaries	Generic	Survey (100 physicians)	Discharge and admission diagnosis contribute most to summary quality	No
What makes a "successful" or "unsuccessful" discharge letter? Hospital clinician and General Practitioner assessments of the quality of discharge letters.	2021	United Kingdo m	To assess what makes a successful or unsuccessful discharge letter from inpatient and outpatient physician perspective	Generic	Mixed-methods (489 discharge letters assessed by 53 GPs) and survey (46 responses)	- Clear diagnosis most important<br>- Include more details in the diagnosis	No
Kurzanleitung zum Schreiben von Arztbriefen		German y	To provide instructions for inpatient physicians on how to write discharge summaries	Generic	Expert opinion	- Provide a clear and concise list of all relevant diagnoses starting with those related to the reason for admission<br>- Group related diagnoses into meaningful blocks (e.g., underlying conditions linked to current issues). Avoid unnecessary sections like “current” or “chronic,” and omit less relevant diagnoses for clarity.<br>- Formulate clear diagnoses, mention hypotheses for unclear symptoms, classify chronic conditions (e.g., severity, duration), and associate current interventions with the relevant diagnoses.<br>- Avoid jargon and use abbreviations only if clearly understandable.	Yes

Review article: Components of a good quality discharge summary: a systematic review.	2014	New Zealand	To identify a consensus on the key components of a high-quality discharge summary	Generic	Systematic (scoping) review	- Make the summary consistent with final diagnosis<br>- List the most important diagnosis first<br>- Include discharge diagnosis as the most important content element	No
Arztbrief: Die Kommunikation optimieren		German y	To explain how communication can be improved in discharge summaries	Generic	Expert opinion	- Include complete and accurate diagnoses, with stages/classification/severity levels<br>- Arrange by clinical relevance, with causally related diagnoses grouped together<br>- List relevant surgeries, potentially under a separate heading	No

Supplementary Fig. 1 Reported usage of the diagnosis section

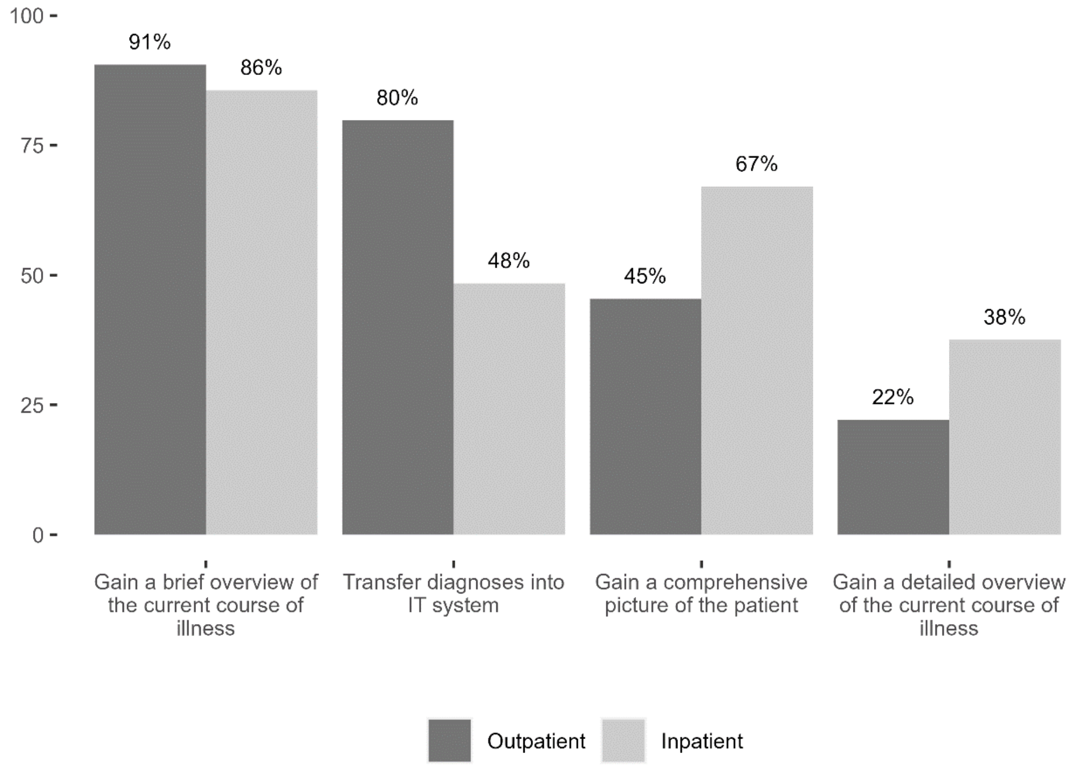

Supplementary Fig. 2 Reasons for insufficient diagnosis sections

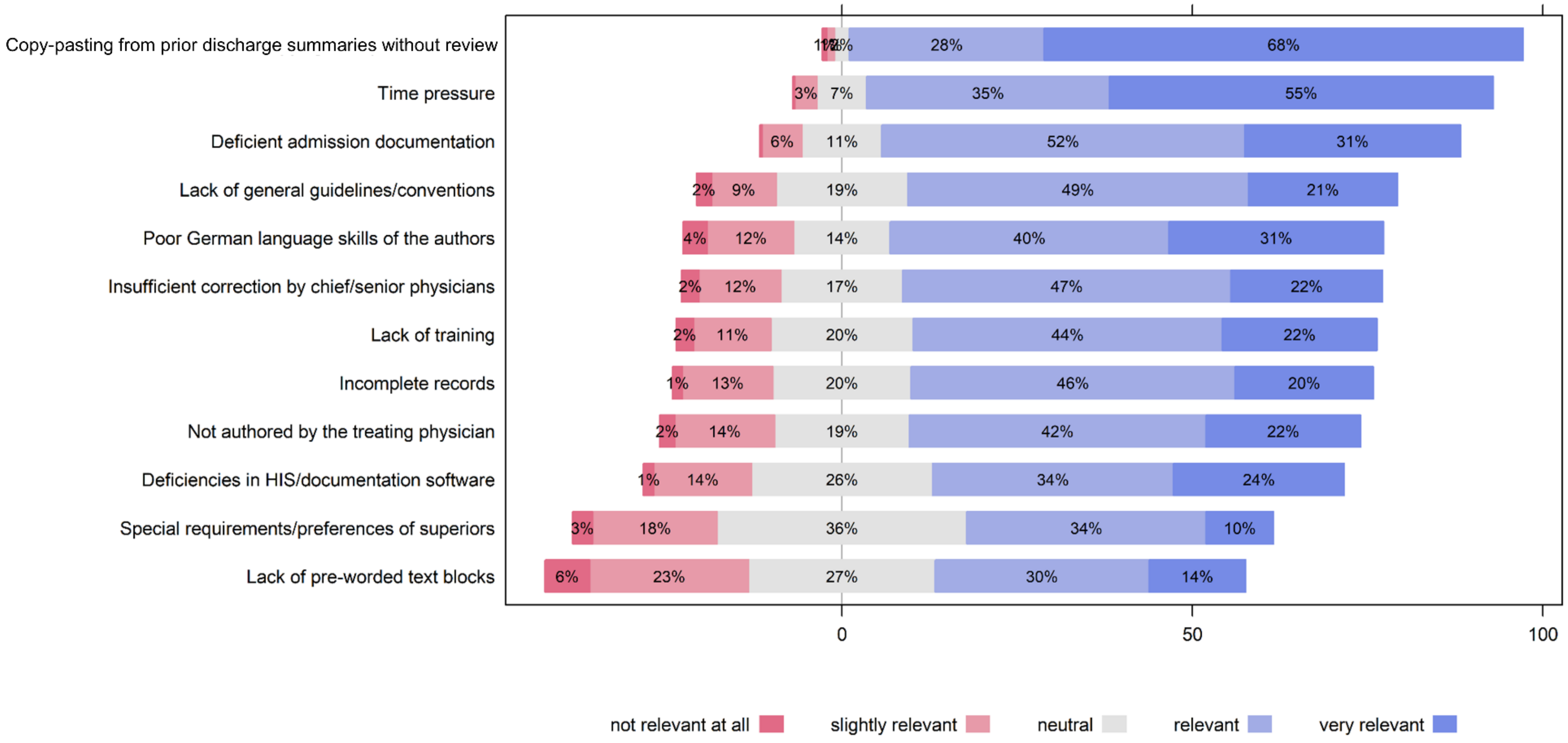

Supplementary Fig. 3 Content elements in the diagnosis section beyond diagnoses

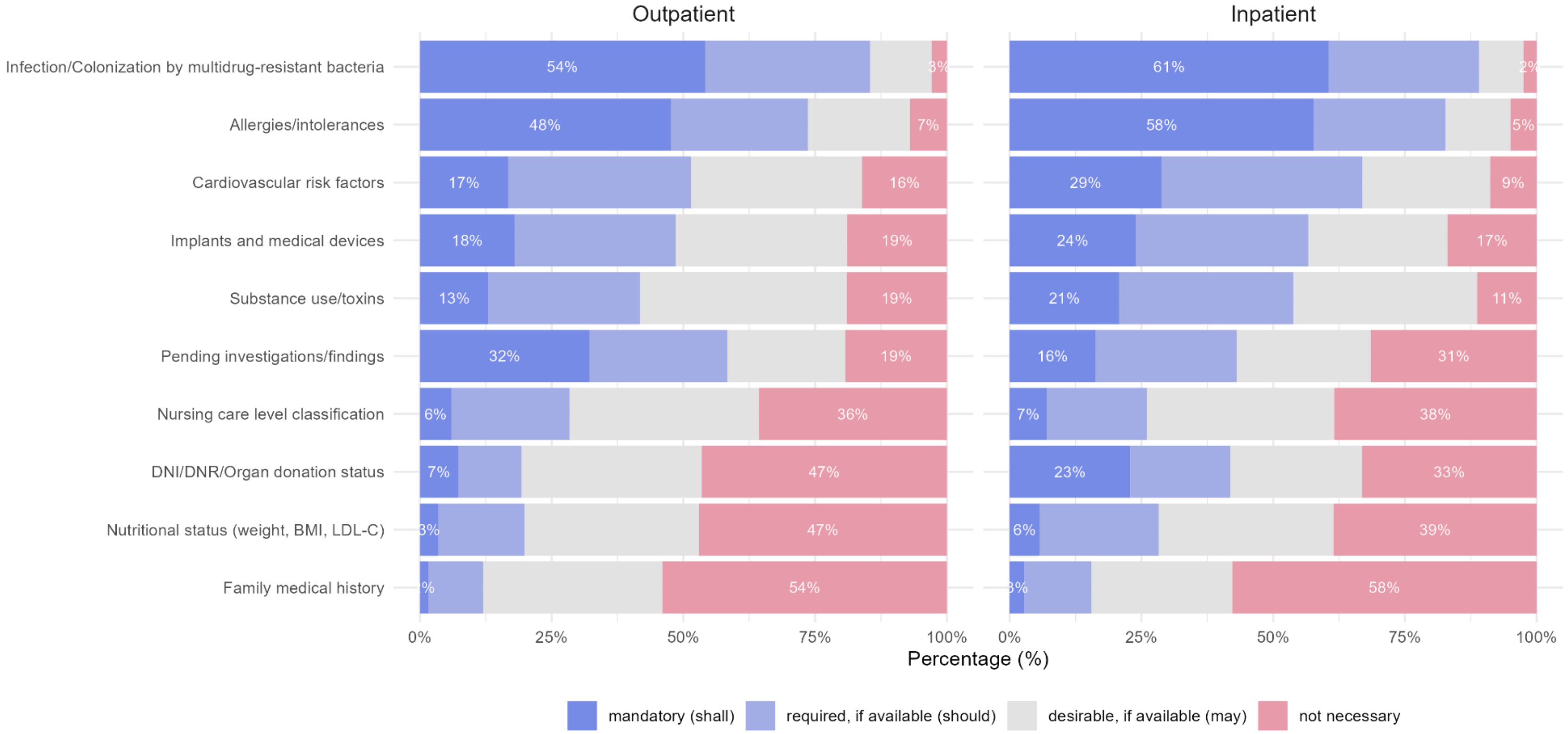

Supplementary Fig. 4 Interactive content elements

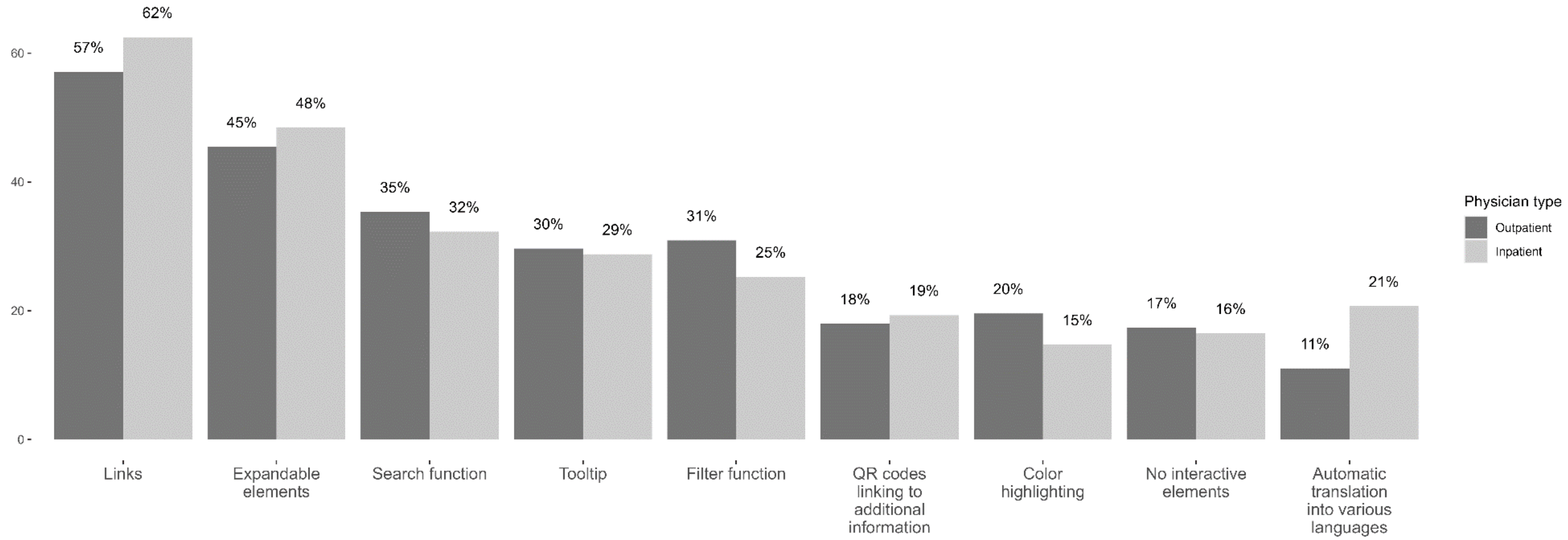

Supplementary Table 8 Structural elements preferred by physicians

		Total (n = 602)		Outpatient (n = 317)		Inpatient (n = 285)	
**Category**	**Preference**	**%**	**Count**	**%**	**Count**	**%**	**Count**
Layout	Paragraphs	52%	313	42%	132	64%	181
	Tabular	46%	274	57%	179	34%	95
	Block text	2%	10	1%	3	2%	7
	No answer	*n/a*	5	*n/a*	3	*n/a*	2
Highlighting relevant information	Highlighting of all important information	42%	249	43%	137	41%	112
	Highlighting the current treatment diagnoses	29%	171	30%	95	28%	76
	Highlighting the word "aktuell"	26%	152	23%	71	29%	81
	"Checkbox" indicating diagnoses treated in current stay	3%	18	4%	12	2%	6
	No answer	*n/a*	12	*n/a*	2	*n/a*	10

Numbering of diagnoses	No numbering	41%	243	30%	95	52%	148
	Numbering only of the current treatment diagnoses	31%	186	36%	112	26%	74
	Continuous numbering	19%	111	25%	78	12%	33
	Separate numbering of current treatment diagnoses and chronic diagnoses	9%	54	9%	27	10%	27
	No answer	*n/a*	8	*n/a*	5	*n/a*	3
Ordering of diagnoses	Clinical relevance	93%	535	94%	289	92%	246
	Body / Organ system	5%	27	3%	10	6%	17
	Etiology / Cause	2%	11	2%	7	1%	4
	Billing relevance	0%	1	0%	0	0%	1
	Alphabetical order	0%	0	0%	0	0%	0
	No answer	*n/a*	28	*n/a*	11	*n/a*	17
Content structuring (headings)	"Current treatment diagnoses", "Chronic diagnoses", and "Past medical history" separated	45%	267	52%	163	37%	104
	Current treatment diagnoses" separated from "Past medical history and chronic diagnoses"	42%	247	39%	124	44%	123
	"Currently" ("Aktuell") under relevant diagnoses	8%	49	6%	18	11%	31
	Everything listed under "Diagnoses"	5%	32	3%	9	8%	23
	No answer	*n/a*	7	*n/a*	3	*n/a*	4
Surgery structuring	Performed procedures / surgeries listed with the diagnoses	49%	280	50%	153	48%	127
	Performed procedures / surgeries under a separate heading	30%	172	25%	76	37%	96
	Performed procedures in parentheses after the diagnosis	20%	115	25%	76	15%	39
	No answer	*n/a*	35	*n/a*	12	*n/a*	23
